# Life satisfaction around the world: Measurement invariance of the Satisfaction With Life Scale (SWLS) across 65 nations, 40 languages, gender identities, and age groups

**DOI:** 10.1371/journal.pone.0313107

**Published:** 2025-01-22

**Authors:** Viren Swami, Stefan Stieger, Martin Voracek, Toivo Aavik, Hamed Abdollahpour Ranjbar, Sulaiman Olanrewaju Adebayo, Reza Afhami, Oli Ahmed, Annie Aimé, Marwan Akel, Hussam Al Halbusi, George Alexias, Khawla F. Ali, Nursel Alp-Dal, Anas B. Alsalhani, Sara Álvarez-Solas, Ana Carolina Soares Amaral, Sonny Andrianto, Trefor Aspden, Marios Argyrides, John Jamir Benzon R. Aruta, Stephen Atkin, Olusola Ayandele, Migle Baceviciene, Radvan Bahbouh, Andrea Ballesio, David Barron, Ashleigh Bellard, Sóley Sesselja Bender, Kerime Derya Beydaǧ, Gorana Birovljević, Marie-Ève Blackburn, Teresita Borja-Alvarez, Joanna Borowiec, Miroslava Bozogáňová, Solfrid Bratland-Sanda, Matthew H. E. M. Browning, Anna Brytek-Matera, Marina Burakova, Yeliz Çakır-Koçak, Pablo Camacho, Vittorio Emanuele Camilleri, Valentina Cazzato, Silvia Cerea, Apitchaya Chaiwutikornwanich, Trawin Chaleeraktrakoon, Tim Chambers, Qing-Wei Chen, Xin Chen, Chin-Lung Chien, Phatthanakit Chobthamkit, Bovornpot Choompunuch, Emilio J. Compte, Jennifer Corrigan, Getrude Cosmas, Richard G. Cowden, Kamila Czepczor-Bernat, Marcin Czub, Wanderson Roberto da Silva, Mahboubeh Dadfar, Simon E. Dalley, Lionel Dany, Jesus Alfonso D. Datu, Pedro Henrique Berbert de Carvalho, Gabriel Lins de Holanda Coelho, Avila Odia S. De Jesus, Sonia Harzallah Debbabi, Sandesh Dhakal, Francesca Di Bernardo, Donka D. Dimitrova, Jacinthe Dion, Barnaby Dixson, Stacey M. Donofrio, Marius Drysch, Hongfei Du, Angel M. Dzhambov, Claire El-Jor, Violeta Enea, Mehmet Eskin, Farinaz Farbod, Lorleen Farrugia, Leonie Fian, Maryanne L. Fisher, Michał Folwarczny, David A. Frederick, Matthew Fuller-Tyszkiewicz, Adrian Furnham, Antonio Alías García, Shulamit Geller, Marta Ghisi, Alireza Ghorbani, Maria Angeles Gomez Martinez, Sarah Gradidge, Sylvie Graf, Caterina Grano, Gyöngyvér Gyene, Souheil Hallit, Motasem Hamdan, Jonathan E. Handelzalts, Paul H. P. Hanel, Steven R. Hawks, Issa Hekmati, Mai Helmy, Tetiana Hill, Farah Hina, Geraldine Holenweger, Martina Hřebíčková, Olasupo Augustine Ijabadeniyi, Asma Imam, Başak İnce, Natalia Irrazabal, Rasa Jankauskiene, Ding-Yu Jiang, Micaela Jiménez-Borja, Verónica Jiménez-Borja, Evan M. Johnson, Veljko Jovanović, Marija Jović, Marko Jović, Alessandra Costa Pereira Junqueira, Lisa-Marie Kahle, Adam Kantanista, Ahmet Karakiraz, Ayşe Nur Karkin, Erich Kasten, Salam Khatib, Nuannut Khieowan, Patricia Joseph Kimong, Litza Kiropoulos, Joshua Knittel, Neena Kohli, Mirjam Koprivnik, Aituar Kospakov, Magdalena Król-Zielińska, Isabel Krug, Garry Kuan, Yee Cheng Kueh, Omar Kujan, Miljana Kukić, Sanjay Kumar, Vipul Kumar, Nishtha Lamba, Mary Anne Lauri, Maria Fernanda Laus, Liza April LeBlanc, Hyejoo J. Lee, Małgorzata Lipowska, Mariusz Lipowski, Caterina Lombardo, Andrea Lukács, Christophe Maïano, Sadia Malik, Mandar Manjary, Lidia Márquez Baldó, Martha Martinez-Banfi, Karlijn Massar, Camilla Matera, Olivia McAnirlin, Moisés Roberto Mebarak, Anwar Mechri, Juliana Fernandes Filgueiras Meireles, Norbert Mesko, Jacqueline Mills, Maya Miyairi, Ritu Modi, Adriana Modrzejewska, Justyna Modrzejewska, Kate E. Mulgrew, Taryn A. Myers, Hikari Namatame, Mohammad Zakaria Nassani, Amanda Nerini, Félix Neto, Joana Neto, Angela Nogueira Neves, Siu-Kuen Ng, Devi Nithiya, Jiaqing O, Sahar Obeid, Camila Oda-Montecinos, Peter Olamakinde Olapegba, Tosin Tunrayo Olonisakin, Salma Samir Omar, Brynja Örlygsdóttir, Emrah Özsoy, Tobias Otterbring, Sabine Pahl, Maria Serena Panasiti, Yonguk Park, Muhammad Mainuddin Patwary, Tatiana Pethö, Nadezhda Petrova, Jakob Pietschnig, Sadaf Pourmahmoud, Vishnunarayan Girishan Prabhu, Vita Poštuvan, Pavol Prokop, Virginia L. Ramseyer Winter, Magdalena Razmus, Taotao Ru, Mirjana Rupar, Reza N. Sahlan, Mohammad Salah Hassan, Anđela Šalov, Saphal Sapkota, Jacob Owusu Sarfo, Yoko Sawamiya, Katrin Schaefer, Michael Schulte-Mecklenbeck, Veya Seekis, Kerim Selvi, Mehdi Sharifi, Anita Shrivastava, Rumana Ferdousi Siddique, Valdimar Sigurdsson, Vineta Silkane, Ana Šimunić, Govind Singh, Alena Slezáčková, Christine Sundgot-Borgen, Gill Ten Hoor, Passagorn Tevichapong, Arun Tipandjan, Jennifer Todd, Constantinos Togas, Fernando Tonini, Juan Camilo Tovar-Castro, Lise Katrine Jepsen Trangsrud, Pankaj Tripathi, Otilia Tudorel, Tracy L. Tylka, Anar Uyzbayeva, Zahir Vally, Edmunds Vanags, Luis Diego Vega, Aitor Vicente-Arruebarrena, Jose Vidal-Mollón, Roosevelt Vilar, Hyxia Villegas, Mona Vintilă, Christoph Wallner, Mathew P. White, Simon Whitebridge, Sonja Windhager, Kah Yan Wong, Eric Kenson Yau, Yuko Yamamiya, Victoria Wai Lan Yeung, Marcelo Callegari Zanetti, Magdalena Zawisza, Nadine Zeeni, Martina Zvaríková, Ulrich S. Tran

**Affiliations:** 1 School of Psychology, Sport and Sensory Sciences, Anglia Ruskin University, Cambridge, United Kingdom; 2 Centre for Psychological Medicine, Perdana University, Kuala Lumpur, Malaysia; 3 Department of Psychology and Psychodynamics, Karl Landsteiner University of Health Sciences, Krems an der Donau, Austria; 4 Faculty of Psychology, Department of Cognition, Emotion, and Methods in Psychology, University of Vienna, Vienna, Austria; 5 Institute of Psychology, University of Tartu, Tartu, Estonia; 6 Department of Psychology, Koç University, Istanbul, Türkiye; 7 Department of Psychology and Behavioural Studies, Ekiti State University, Ado-Ekiti, Nigeria; 8 Department of Art Studies, Tarbiat Modares University, Tehran, Iran; 9 Department of Psychology, University of Chittagong, Chattogram, Bangladesh; 10 Department of Psychoeducation and Psychology, Université du Québec en Outaouais, Saint-Jérôme, Canada; 11 INSPECT-LB: National Institute of Public Health, Clinical Epidemiology and Toxicology, Beirut, Lebanon; 12 School of Pharmacy, Lebanese International University, Beirut, Lebanon; 13 Department of Management, Ahmed Bin Mohammad Military College, Doha, Qatar; 14 Faculty of Psychology, Panteion University of Social and Political Sciences, Athens, Greece; 15 Royal College of Surgeons Ireland-Bahrain, Adliya, Bahrain; 16 Faculty of Health Sciences, Department of Midwifery, Munzur University, Tunceli, Türkiye; 17 Department of Oral Medicine and Diagnostic Sciences, Vision College of Dentistry and Nursing, Vision Colleges, Riyadh, Saudi Arabia; 18 Facultad de Ciencias de la Vida, Universidad Regional Amazónica Ikiam, Muyuna, Ecuador; 19 Federal Institute of Education, Science, and Technology of Southeast of Minas Gerais, Barbacena, Brazil; 20 Department of Psychology, Universitas Islam Indonesia, Yogyakarta, Indonesia; 21 Department of Psychology, Aberystwyth University, Ceredigion, United Kingdom; 22 Department of Psychology, Neapolis University Pafos, Paphos, Cyprus; 23 Department of Psychology, De La Salle University, Manila, Philippines; 24 Department of General Studies, The Polytechnic, Ibadan, Nigeria; 25 Department of Psychology, University of Ibadan, Ibadan, Nigeria; 26 Faculty of Health Sciences, Health Research and Innovation Science Centre, Klaipeda University, Klaipeda, Lithuania; 27 Faculty of Arts, Department of Psychology, Charles University, Prague, Czechia; 28 Department of Psychology, Sapienza University of Rome, Rome, Italy; 29 School of Social Sciences, Heriot-Watt University Malaysia, Putrajaya, Malaysia; 30 Faculty of Health, School of Psychology, Liverpool John Moores University, Liverpool, United Kingdom; 31 Faculty of Nursing and Midwifery, University of Iceland, Reykjavik, Iceland; 32 Department of Nursing, Faculty of Health Sciences, Yalova, University, Yalova, Türkiye; 33 Department of Psychology, Faculty of Humanities and Social Sciences, University of Rijeka, Rijeka, Croatia; 34 ÉCOBES-Research and Transfer, Cégep de Jonquière, Québec, Canada; 35 Colegio de Ciencias Sociales y Humanidades, Universidad San Francisco de Quito USFQ, Quito, Ecuador; 36 Department of Physical Education and Lifelong Sports, Poznań University of Physical Education, Poznań, Poland; 37 Institute of Social Sciences of the Centre of Social and Psychological Sciences, Košice, Slovakia; 38 Faculty of Humanities and Natural Sciences, Institute of Pedagogy, Andragogy, and Psychology, University of Prešov, Prešov, Slovakia; 39 Department of Sports, Physical Education and Outdoor Studies, University of South-Eastern Norway, Bø in Telemark, Norway; 40 Department of Parks, Recreation and Tourism Management, Clemson University, Clemson, South Carolina, United States of America; 41 Institute of Psychology, University of Wrocław, Wrocław, Poland; 42 Laboratory of Social Psychology, Aix-Marseille University, Aix-en-Provence, France; 43 Department of Midwifery, Faculty of Health Sciences, Bartın University, Bartın, Türkiye; 44 CentroSan Isidoro University Center, Seville, Spain; 45 Department of Psychology, University of Malta, Msida, Malta; 46 Department of Cognitive Sciences, Psychology, Education, and Cultural Studies, University of Messina, Messina, Italy; 47 Department of General Psychology, University of Padova, Padova, Italy; 48 Department of Biomedical Sciences, University of Padova, Padova, Italy; 49 Faculty of Psychology, Chulalongkorn University, Bangkok, Thailand; 50 Faculty of Liberal Arts, Department of Psychology, Thammasat University, Pathumthani, Thailand; 51 School of Psychology, Deakin University, Geelong, Australia; 52 Lab of Light and Physio-Psychological Health, National Center for International Research on Green Optoelectrics, South China Normal University, Guangzhou, China; 53 Guangdong Provincial Key Laboratory of Optical Information Materials and Technology & Institute of Electronic Paper Displays, South China Academy of Advanced Optoelectronics, South China Normal University, Guangzhou, China; 54 Department of Psychology, Graduate School of Arts and Science, New York University, New York, New York, United States of America; 55 Department of Psychology, Soochow University, Taipei, Taiwan; 56 Faculty of Education, Department of Educational Psychology and Guidance, Mahasarakham University, Maha Sarakham, Thailand; 57 School of Psychology, Universidad Adolfo Ibáñez, Penalolen, Chile; 58 Comenzar de Nuevo Treatment Center, Monterrey, México; 59 School of Applied Psychology, University College Cork, Cork, Ireland; 60 Faculty of Psychology and Education, Universiti Malaysia Sabah, Kota Kinabalu, Malaysia; 61 Human Flourishing Program, Harvard University, Cambridge, Massachusetts, United States of America; 62 Faculty of Medical Sciences in Katowice, Department of Pediatrics, Pediatric Obesity and Metabolic Bone Diseases, Medical University of Silesia, Katowice, Poland; 63 Graduate Program in Food, Nutrition and Food Engineering, São Paulo State University, São Paulo, Brazil; 64 Department of Addiction, School of Behavioral Sciences and Mental Health (Tehran Institute of Psychiatry), Iran University of Medical Sciences, Tehran, Iran; 65 Department of Psychology, University of Groningen, Groningen, The Netherlands; 66 Faculty of Education, Academic Unit of Human Communication, Learning, and Development, The University of Hong Kong, Hong Kong, China; 67 Body Image and Eating Disorders Research Group, Federal University of Juiz de Fora, Juiz de Fora, Brazil; 68 Institute of Psychiatry, University of São Paulo, São Paulo, Brazil; 69 Faculty of Medicine, University of Sousse, Sousse, Tunisia; 70 Central Department of Psychology, Tribhuvan University, Kathmandu, Nepal; 71 Department of Psychology, University of Campania Luigi Vanvitelli, Caserta, Italy; 72 Faculty of Public Health, Department of Health Management and Healthcare Economics, Medical University of Plovdiv, Plovdiv, Bulgaria; 73 Environmental Health Division, Research Institute at Medical University of Plovdiv, Medical University of Plovdiv, Plovdiv, Bulgaria; 74 Department of Psychology, Université du Québec à Trois-Rivières, Trois-Rivières, Canada; 75 School of Health, University of the Sunshine Coast, Moreton Bay, Australia; 76 Department of Plastic and Hand Surgery, BG University Hospital Bergmannsheil Bochum, Ruhr University Bochum, Bochum, Germany; 77 Department of Psychology, Beijing Normal University at Zhuhai, Zhuhai, China; 78 Health and Quality of Life in a Green and Sustainable Environment Research Group, Strategic Research and Innovation Program, Medical University of Plovdiv, Plovdiv, Bulgaria; 79 Department of Natural Sciences, School of Arts and Sciences, Lebanese American University, Beirut, Lebanon; 80 Department of Psychology, Alexandru Ioan Cuza University, Iași, Romania; 81 Department of Psychology, Koc University, Istanbul, Türkiye; 82 Department of Textile and Fashion Design, Alzahra University, Tehran, Iran; 83 Department of Psychology, Saint Mary’s University, Halifax, Canada; 84 Discipline of Marketing, J.E. Cairnes School of Business & Economics, University of Galway, Galway, Ireland; 85 Crean College of Health and Behavioral Sciences, Chapman University, Orange, California, United States of America; 86 Department of Leadership and Organizational Behaviour, Norwegian Business School, Oslo, Norway; 87 Department of Education, University of Almería, Almería, Spain; 88 School of Behavioral Sciences, The Academic College of Tel Aviv-Yaffo, Tel Aviv-Yafo, Israel; 89 Unità Operativa Complessa (UOC) Hospital Psychology, Padova University Hospital, Padova, Italy; 90 Department of Social Sciences, Payam Noor University, Tehran, Iran; 91 Faculty of Psychology, Pontifical University of Salamanca, Salamanca, Spain; 92 Institute of Psychology, Czech Academy of Sciences, Brno, Czechia; 93 Doctoral School of Psychology, Eötvös Loránd University, Budapest, Hungary; 94 School of Medicine and Medical Sciences, Holy Spirit University of Kaslik, Jounieh, Lebanon; 95 Applied Science Research Center, Applied Science Private University, Amman, Jordan; 96 Faculty of Public Health, Al-Quds University, East Jerusalem, Palestine; 97 Department of Psychiatry, University of Michigan, Ann Arbor, Michigan, United States of America; 98 Department of Psychology, University of Essex, Colchester, United Kingdom; 99 Department of Kinesiology and Health Science, Utah State University, Logan, Utah, United States of America; 100 Department of Psychology, Faculty of Human Science, University of Maragheh, Maragheh, Iran; 101 Department of Psychology, College of Education, Sultan Qaboos University, Muscat, Oman; 102 Department of Psychology, Faculty of Arts, Menoufia University, Shebin el Kom, Egypt; 103 Hertfordshire Business School, University of Hertfordshire, Hatfield, United Kingdom; 104 Department of Psychiatry, University of Cambridge, Cambridge, United Kingdom; 105 Department of Consumer Behavior, University of Bern, Bern, Switzerland; 106 Department of Sociology and Social Justice, Afe Babalola University, Ado-Ekiti, Nigeria; 107 Department of Psychological Medicine, Institute of Psychiatry, Psychology, and Neuroscience, Centre for Research in Eating and Weight Disorders, King’s College London, London, United Kingdom; 108 Faculty of Social Sciences, University of Palermo, Buenos Aires, Argentina; 109 Department of Psychology, National Chung Cheng University, Chia Yi, Taiwan; 110 Colegio de Comunicación y Artes Contemporáneas, Universidad San Francisco de Quito USFQ, Quito, Ecuador; 111 Department of Psychology, Faculty of Philosophy, University of Novi Sad, Novi Sad, Serbia; 112 Faculty of Organizational Sciences, University of Belgrade, Belgrade, Serbia; 113 Faculty of Medicine, University of Belgrade, Belgrade, Serbia; 114 Department of Psychology, University of São Paulo, Ribeirão Preto, Brazil; 115 Department of Nutrition, University of Ribeirão Preto, Ribeirão Preto, Brazil; 116 Faculty of Life Sciences, Medical School Hamburg, Hamburg, Germany; 117 Sakarya Business School, Sakarya University, Sakarya, Türkiye; 118 Practice for Psychotherapy, Am Krautacker 25, Travemünde, Germany; 119 Faculty of Health Professions, Al-Quds University, East Jerusalem, Palestine; 120 Asian Studies Department, Faculty of International Studies, Prince of Songkla University Phuket Campus, Phuket, Thailand; 121 School of Psychological Sciences, University of Melbourne, Melbourne, Australia; 122 Department of Psychology, University of Allahabad, Prayagraj, India; 123 Institute of Anton Martin Slomsek, Primary School Montessori, Maribor, Slovenia; 124 Department of Sociology and Social Work, Al-Farabi Kazakh National University, Almaty, Kazakhstan; 125 Department of General Education Disciplines, Astana IT University, Astana, Kazakhstan; 126 Exercise and Sport Sciences Programme, School of Health Sciences, Universiti Sains Malaysia, Kubang Kerian, Malaysia; 127 Biostatistics and Research Methodology Unit, School of Medical Sciences, Universiti Sains Malaysia, Kubang Kerian, Malaysia; 128 Oral Diagnostics and Surgical Sciences, UWA Dental School, The University of Western Australia, Nedlands, Australia; 129 Department of Psychology, D.A.V. College, Muzaffarnagar, India; 130 Department of Psychology, Kashi Naresh Government Post-Graduate College, Gyanpur, India; 131 Department of Psychology, Middlesex University Dubai, Dubai, United Arab Emirates; 132 Department of Counselling Psychology and Social Welfare, Handong Global University, Pohang, South Korea; 133 Institute of Psychology, University of Gdańsk, Gdańsk, Poland; 134 Faculty of Social and Humanities, University WSB Merito, Gdansk, Poland; 135 Faculty of Health Sciences, University of Miskolc, Miskolc, Hungary; 136 Cyberpsychology Laboratory, Department of Psychoeducation and Psychology, Université du Québec en Outaouais (UQO), Saint-Jérôme, Canada; 137 Department of Psychology, University of Sargodha, Sargodha, Pakistan; 138 M.M.D. Public School, Brahmpuri, Muzaffarnagar, India; 139 Department of Research Methods and Diagnosis in Education, University of València, València, Spain; 140 Faculty of Legal and Social Sciences, Simón Bolívar University, Barranquilla, Colombia; 141 Life Science Research Centre, Simón Bolívar University, Barranquilla, Colombia; 142 Department of Work and Social Psychology, Maastricht University, Maastricht, The Netherlands; 143 Department of Education, Languages, Intercultures, Literatures, and Psychology, University of Florence, Florence, Italy; 144 Department of Psychology, Universidad del Norte, Barranquilla, Colombia; 145 Faculty of Medicine of Monastir, Eya Medical Centre, Monastir, Tunisia; 146 Department of Family and Community Medicine, School of Community Medicine, University of Oklahoma, Tulsa, Oklahoma, United States of America; 147 Institute of Psychology, University of Pécs, Pécs, Hungary; 148 Department of Health Sciences, DePaul University, Chicago, Illinois, United States of America; 149 Faculty of Medical Sciences in Katowice, Department of Medical Anthropology, Medical University of Silesia, Katowice, Poland; 150 Institute of Pedagogy, University of Bielsko-Biala, Bielsko-Biala, Poland; 151 Department of Psychology, Virginia Wesleyan University, Virginia Beach, Virginia, United States of America; 152 Faculty of Human Sciences, University of Tsukuba, Tsukuba, Japan; 153 Department of Restorative and Prosthetic Dental Sciences, College of Dentistry, Dar Al Uloom University, Riyadh, Saudi Arabia; 154 Faculty of Psychology and Educational Sciences, University of Porto, Porto, Portugal; 155 REMIT–Research on Economics, Management and Information Technologies, Universidade Portucalense, Porto, Portugal; 156 Division of Research, Physical Education College of the Brazilian Army, Rio de Janeiro, Brazil; 157 Physical Education Unit, Chinese University of Hong Kong, Hong Kong, China; 158 Department of Physiology, Mahatma Gandhi Medical College and Research Institute, Puducherry, India; 159 Social and Education Sciences Department, School of Arts and Sciences, Lebanese American University, Jbeil, Lebanon; 160 Institute of Social Sciences, Universidad de O’Higgins, Rancagua, Chile; 161 Department of Dermatology, Venereology, and Andrology, Alexandria University, Alexandria, Egypt; 162 Department of Management, University of Agder, Kristiansand, Norway; 163 Scientific Institute for Research and Healthcare, Santa Lucia Foundation, Rome, Italy; 164 Department of Psychology, Kyungnam University, Changwon, South Korea; 165 Environment and Sustainability Research Initiative, Khulna, Bangladesh; 166 Environmental Science Discipline, Life Science School, Khulna University, Khulna, Bangladesh; 167 Faculty of Medicine, Department of Human Anatomy, Histology and Embryology, Medical University of Plovdiv, Plovdiv, Bulgaria; 168 Faculty of Psychology, Department of Developmental and Educational Psychology, University of Vienna, Vienna, Austria; 169 Industrial and Systems Engineering, University of North Carolina, Charlotte, North Carolina, United States of America; 170 Slovene Centre for Suicide Research, Andrej Marusic Institute, University of Primorska, Koper, Slovenia; 171 Department of Psychology FAMNIT, University of Primorska, Koper, Slovenia; 172 Faculty of Natural Sciences, Department of Environmental Ecology and Landscape Management, Comenius University in Bratislava, Bratislava, Slovakia; 173 Institute of Zoology, Slovak Academy of Sciences, Bratislava, Slovakia; 174 School of Social Work, University of Missouri, Columbia, Missouri, United States of America; 175 Institute of Psychology, Marie Curie-Skłodowska University, Lublin, Poland; 176 Department of Counseling, School, and Educational Psychology, Graduate School of Education, University at Buffalo-SUNY, Buffalo, New York, United States of America; 177 Management Department, College of Business Administration, A’Sharqiyah University, Ibra, Oman; 178 Department of Psychology, University of Zadar, Zadar, Croatia; 179 KOSHISH-National Mental Health Self-Help Organization, Kusunti, Lalitpur, Nepal; 180 Department of Health, Physical Education and Recreation, University of Cape Coast, Cape Coast, Ghana; 181 Department of Evolutionary Anthropology, University of Vienna, Vienna, Austria; 182 Human Evolution and Archaeological Sciences, University of Vienna, Vienna, Austria; 183 Max Planck Institute for Human Development, Berlin, Germany; 184 School of Applied Psychology, Griffith University, Gold Coast, Australia; 185 Department of Psychology, Eskişehir Osmangazi University, Eskişehir, Türkiye; 186 Department of Psychology, Islamic Azad University, Bandar Gaz, Iran; 187 Department of Psychology, University of Dhaka, Dhaka, Bangladesh; 188 Department of Business Administration, Reykjavik University, Reykjavik, Iceland; 189 Faculty of Social Sciences, Vidzeme University of Applied Sciences, Valmiera, Latvia; 190 Faculty of Medicine, Department of Medical Psychology and Psychosomatics, Masaryk University, Brno, Czechia; 191 Regional Department for Eating Disorders, Oslo University Hospital, Oslo, Norway; 192 Faculty of Humanities, Department of Psychology, Chiang Mai University, Chiang Mai, Thailand; 193 International Centre for Psychological Counselling and Social Research, Puducherry, India; 194 Department of Psychology, University of the Andes, Bogotá, Colombia; 195 Department of Psychology, West University of Timişoara, Timişoara, Romania; 196 Department of Psychology, The Ohio State University, Ohio, Columbus, United States of America; 197 Department of Clinical Psychology, United Arab Emirates University, Al Ain, United Arab Emirates; 198 Faculty of Education, Psychology, and Art, University of Latvia, Rīga, Latvia; 199 Vice-rectory for Teaching, Research, and Extension, Universida Latina de Costa Rica, San José, Costa Rica; 200 Department of Psychology, Massey University, Auckland, New Zealand; 201 School of Psychology, University of Nottingham Malaysia, Semenyih, Malaysia; 202 Department of Psychology, Lingnan University, Hong Kong, China; 203 Department of Undergraduate Studies, Temple University, Japan Campus, Tokyo, Japan; 204 Wofoo Joseph Lee Consulting and Counselling Psychology Research Centre, Lingnan University, Hong Kong, China; 205 Department of Physical Education, São Judas Tadeu University, São Paulo, Brazil; The Open University of Israel, ISRAEL

## Abstract

The Satisfaction With Life Scale (SWLS) is a widely used self-report measure of subjective well-being, but studies of its measurement invariance across a large number of nations remain limited. Here, we utilised the Body Image in Nature (BINS) dataset–with data collected between 2020 and 2022 –to assess measurement invariance of the SWLS across 65 nations, 40 languages, gender identities, and age groups (*N* = 56,968). All participants completed the SWLS under largely uniform conditions. Multi-group confirmatory factor analysis indicated that configural and metric invariance was upheld across all nations, languages, gender identities, and age groups, suggesting that the unidimensional SWLS model has universal applicability. Full scalar invariance was achieved across gender identities and age groups. Based on alignment optimisation methods, partial scalar invariance was achieved across all but three national groups and across all languages represented in the BINS. There were large differences in latent SWLS means across nations and languages, but negligible-to-small differences across gender identities and age groups. Across nations, greater life satisfaction was significantly associated with greater financial security and being in a committed relationship or married. The results of this study suggest that the SWLS largely assesses a common unidimensional construct of life satisfaction irrespective of respondent characteristics (i.e., national group, gender identities, and age group) or survey presentation (i.e., survey language). This has important implications for the assessment of life satisfaction across nations and provides information that will be useful for practitioners aiming to promote subjective well-being internationally.

## Introduction

The construct of *subjective well-being* generally refers to the ways in which individuals think and experience their lives in positive versus negative ways [1] and has been conceptualised as being multifaceted, with both affective and cognitive components [2]. Among the constituent components of subjective well-being is the construct of *life satisfaction*, a cognitive, judgemental process in which “individuals assess the quality of their lives on the basis of their own unique set of criteria” (p. 164) [3]. Life satisfaction is associated with, but distinct from, other facets of subjective well-being (e.g., positive affect) [4,5], and is positively related to indices across a broad range of life domains, including overall mortality, physical health functioning, and occupational outcomes [6–9]. As such, it is vital that researchers and practitioners have appropriate tools to measure the construct of life satisfaction in diverse communities [5].

Although various single- and multi-item instruments have been developed to measure life satisfaction [10], by far the most widely used is the Satisfaction With Life Scale (SWLS) [11]. The SWLS consists of five items, three of which generally refer to present life satisfaction (Items #1, 2, and 3) and two to past life satisfaction (Items #4 and 5). Originally developed with college-aged and elderly samples in the United States as an assessment of an individual’s global judgement of their life satisfaction [11], the SWLS is now widely used in adult populations [12,13] and increasingly used in adolescent [14] and clinical populations [15]. Indeed, the original report on the SWLS [10] has received almost 47,000 citations to date (based on Google Scholar citations up to September 2024). One reason for this wide usage is the wealth of evidence supporting the factorial validity of SWLS scores, with studies strongly supporting unidimensionality and adequacy of composite reliability [5,13,16,17] using a range of statistical approaches [18]. A large body of evidence also supports the nomological network of SWLS scores, including its convergent and divergent validity [5,13].

The unidimensionality of SWLS scores has also been extensively supported in diverse national and social identity groups from many world regions [19–22]. Nevertheless, studies in singular national and/or cultural contexts still leaves unanswered the question of whether the SWLS measures the same latent construct of life satisfaction across diverse groups (i.e., the extent to which SWLS scores achieve *measurement invariance*) [23]. This is important because many scholars consider measurement invariance to be a prerequisite of any meaningful comparison of latent scores, as well as examination of differential relations between constructs, across groups [24–26]. Where the measurement properties of an instrument fundamentally differ across two or more groups, measurement biases could occur, leading to artefactual, inaccurate, or irreplicable results [27]. Measurement invariance can be determined at different levels, including configural (i.e., invariance of model form), metric (i.e., equivalence of item loadings on factors), scalar (i.e., equivalence of item intercepts), and strict levels (i.e., equivalence of item residuals of metric and scalar invariance items) of invariance [23,28]. Scalar or partial scalar invariance is typically considered a minimum threshold for comparison of latent means [29,30].

Studies that have assessed measurement invariance of the SWLS across national groups have returned somewhat equivocal results. Some studies that have assessed measurement invariance across two [31,32], three [33–35], or five nations [36]–primarily in South America and Western Europe, and typically with university samples that are not often representative of broader populations–have reported evidence supporting invariance through to full or partial scalar invariance. However, most studies–the majority of which have assessed invariance across two nations–have failed to find any support for scalar invariance. For instance, one review of studies published between 1985 and 2016 and reporting predominantly on European and Asian datasets, found that 10 of 11 eligible studies supported only configural or metric invariance [37]. Other bi-national comparisons (mainly across Latin America and Spain) not included in the review have similarly been equivocal, with some supporting full scalar invariance [38] and others supporting only metric invariance [39]. Importantly, non-invariance of measurement parameters appears to affect each of the five SWLS items [37], suggesting that none of the SWLS items are measuring the construct of life satisfaction in the same way across nations.

Notably, however, studies across two to five national groups predominantly in South America, Western Europe, and Asia offer only a piecemeal evaluation of measurement invariance. To date, only two studies have assessed measurement invariance of the SWLS across a larger number of nations. In the first, Jang and colleagues [40] found support for partial scalar invariance in samples of managers from 26 nations (11 in Europe, 6 in Asia, 4 in South America, 3 in North America, and 2 in Australasia) once the intercepts for three SWLS items (Items #2, 4, and 5) were relaxed. In the second, Jovanović et al. [41] examined measurement invariance in samples of adolescents from 24 nations (13 in Europe, 9 in Asia, and 1 each in Africa and South America). Their results indicated support for configural invariance across 19 nations but only when including residual covariance between two items (Items #4 and 5). Using the same model, partial scalar invariance was supported across nine nations, whereas partial metric invariance was supported across all 24 nations when allowing for different pairs of item residuals to covary. Other relevant work has likewise suggested that the distinction between items referring to present (i.e., Items #1, 2, and 3) and past life satisfaction (i.e., Items #4 and 5) may be a source of non-invariance [12,42–44].

It is possible that understandings and construals of global cognitive judgements about life satisfaction vary widely across nations, which may help explain the aforementioned difficulties achieving scalar invariance on the SWLS. That is, what it means to be satisfied with one’s life is likely embedded within specific national contexts and/or within language structures [45,46] and hence may result in differential functioning of SWLS items across nations [41,47] based on what respondents bring to mind when responding to specific SWLS items [48]. Some support for this suggestion comes from qualitative research comparing understandings of life satisfaction across national groups. For example, when participants were asked to consider definitions of life satisfaction and discuss specific SWLS items, respondents from Germany were more focused on the fulfilment of basic needs (e.g., a place to live), whereas Chinese participants referred to good living conditions (e.g., high income) [49]. Similarly, while life satisfaction was predominantly couched in terms of adjustment and contentment among elderly men in the United Kingdom, it was more closely related to filial closeness among elderly men in India [50]. These studies suggest that the non-invariance of SWLS items may, in part, be grounded in national or cultural differences in the conception, experience, or manifestation of life satisfaction.

As with invariance across national groups, studies examining invariance across other demographic characteristics, such as gender identities and age, have also returned mixed findings. For instance, of the 14 eligible studies that assessed gender invariance and that were included in the review by Emerson et al. [37], eight supported scalar or strict invariance, whereas six were only able to support configural or metric invariance. Likewise, of nine studies that assessed invariance across age groups (i.e., across age quintiles or across stages of adulthood), only one supported scalar invariance, whereas the remaining eight supported configural or metric invariance, or failed to support any form of invariance [37]. Notably, however, more recent studies in singular nations (e.g., the United States, South Korea) not included in the review have generally supported scalar invariance across both gender and age quintiles [32,51–53], typically once the unidimensional model of SWLS scores is modified to include residual covariance between Items #4 and 5 (i.e., the two items referring to past life satisfaction) [41,54]. Nevertheless, it remains possible that social and/or cultural conditions that yield different gendered and age-related experiences result in varying understandings of the meaning or phenomenology of life satisfaction as measured by the SWLS [49,55].

### Life satisfaction around the world

Large, multinational studies offer the best opportunities to (re-)consider issues of invariance *vis-à-vis* the SWLS, but existing studies have either relied on predicted or simulated data [56,57], or have relied on samples from limited world regions, which may mean that findings with regards to invariance were driven by sample characteristics (e.g., homogeneity of cultural background, particularly when relying on university samples) rather than the construct of life satisfaction. Additionally, existing studies may have also been hampered by practical considerations that may have affected aspects of invariance. For instance, the study by Jang et al. [40] recruited predominantly male company managers, with some within-nation subsample sizes possibly being under-powered (within country sample sizes ranged from 157 to 500). Similarly, Jovanović et al. [41] utilised both primary and secondary data spanning a decade (2010–2020), with methods of participant recruitment and survey completion varying across nations. Although such issues may seem trivial, aspects of operational equivalence (i.e., the characteristics of using an instrument in different populations) can influence how items on an instrument are understood and completed [58], which in turn may shape the response characteristics of items [59]. Ensuring commonality of operational practices, insofar as possible in multinational studies, is therefore an important prerequisite for assessments of measurement invariance [26,60].

There are additional reasons why it may be important to assess life satisfaction in a cross-national setting at this time. First, populations globally have been dealing with the coronavirus disease 2019 (COVID-19) pandemic, which may have impacted life satisfaction [61,62]. For instance, pandemic-specific experiences (e.g., COVID-19 perceived risk, fear of COVID-19, resource loss) have been implicated as determinants of psychological well-being during the pandemic [63–66], though such experiences are unlikely to have been uniform across diverse social identity groups. Indeed, one study showed that life satisfaction, and the predictors of life satisfaction, varied between university students in nine (mainly European) nations during the first wave of the pandemic–although notably, these authors appear to have assumed invariance of SWLS scores [67]. In light of such findings, as well as work showing that high life satisfaction buffered against symptoms of depression, anxiety, and stress during the pandemic [68,69], it would be useful to better understand cross-national differences and/or similarities in life satisfaction during the period of the COVID-19 pandemic [70], which should be predicated upon measurement invariance [56].

Second, a deeper understanding of correlates of life satisfaction across nations could provide useful indicators of the ways in which life satisfaction measures could informatively shape public policy and practice [71,72]. For instance, it would be useful to more fully examine sociodemographic correlates of life satisfaction and the stability of such associations across nations. Thus, an influential strand of research on subjective well-being indicates that the fulfilment of basic human needs and greater material or financial well-being is associated with greater life satisfaction [73–76]. Likewise, it has been suggested that those in committed relationships may be more likely to experience greater life satisfaction than those who are unpartnered, possibly because the formation and maintenance of close social relationships provides greater opportunities to support psychological well-being [77,78]. While both of these reported associations may be supported theoretically and empirically [79,80], they have generally not been tested across a large number of nations, which may be important because understandings and conceptions of material well-being and social support may vary across cultures.

Additionally, reported associations between other sociodemographic factors and life satisfaction tend to be more equivocal. For example, multinational studies that have assessed differences in life satisfaction or psychological well-being more generally across respondents living in urban and rural areas (e.g., in the European Union) have either reported no significant differences [81] or a small difference in favour of rural respondents [82]. Likewise, studies in singular nations have variously reported that urban respondents [79] or rural respondents [83,84] have greater life satisfaction or psychological well-being more generally, although there is high variability both between and across rural and urban communities [85]. Finally, studies examining differences in life satisfaction as a function of racialised majority or minority status have also been equivocal [74,86–88] and limited to singular nations. Overall, then, a better understanding of the way in which sociodemographic variables are associated with life satisfaction, especially in a cross-national context, is necessary to better facilitate strategies to improve life satisfaction across and within nations [89].

### The present study

To summarise, with few exceptions [40,41], existing research has infrequently assessed measurement invariance of the SWLS across a large number of national groups, languages, gender identities, and age groups. Indeed, scholars have often bemoaned the lack of availability of global datasets that would allow for improved tests of measurement invariance [56]. Likewise, with notable exceptions [67], there is limited information about cross-national stability of sociodemographic correlates of SWLS scores. To address these issues, we utilised data from the Body Image in Nature Survey (BINS) [90], a collaborative, researcher-crowdsourced project that gathered cross-sectional SWLS data between 2020 and 2022 from participants in 65 nations. The BINS dataset presents unprecedented opportunities to advance knowledge in a number of ways, including examination of invariance across a larger set of nations than in any earlier study, examination of (for the first time) invariance across a diverse set of languages, and assessment of life satisfaction on a global stage during the period of COVID-19 pandemic.

Based on the findings of previous large, multinational studies [40,41], we expected to obtain evidence of at least partial scalar invariance of the SWLS across nations once residual covariance between Items #4 and 5 has been accounted for. Likewise, we also expected to demonstrate full or partial scalar invariance across gender identities and age groups (i.e., emerging adults: 18–24 years; young adults: 25–44 years; middle-age and older adults: ≥ 45 years) [91,92]. As a novel and exploratory extension to this literature, and based on the suggestion that linguistic structures may affect responding to life satisfaction items [45], we also examined invariance across the 40 languages represented in the BINS. Should full or partial scalar invariance be established, this would offer an unprecedented opportunity to examine differences in latent SWLS scores across many nations, languages, gender identities, and age groups. In addition to examining latent differences, the BINS dataset also allowed us to examine sociodemographic correlates of life satisfaction. Specifically, we preliminarily hypothesised that, across nations, greater life satisfaction would be significantly associated with self-identifying as part of a racialised majority, residing in rural areas, higher education (a proxy of material well-being), being married or in a relationship, and greater financial security.

## Materials and methods

### Overview of the body image in nature survey

The Body Image in Nature Survey (BINS) is a researcher-crowdsourced project involving 253 scientists working collaboratively across 65 nations (for a detailed, published study protocol, see [90]). All cross-sectional data were collected between November 2020 and February 2022 via community sampling, with the majority of recruitment taking place online. In practice, this meant that an online survey was available for completion by participants who met inclusion criteria or, in a small number of sites, researchers directly recruited participants to complete a paper-and-pencil survey. The overall project received ethics approval from the School Research Ethics Panel at the first author’s institution (approval code: PSY-S19-015) and, unless exempt by national laws, all collaborating teams additionally obtained ethics approval from local institutional ethics committees or review boards. A list of nations, associated sample sizes, data collection methods, ethics approvals, and survey languages is presented in S1 Table.

### Participants

The BINS dataset consists of 56,968 respondents from 65 nations, of whom 58.9% (*n* = 33,539) were women, 40.5% (*n* = 23,083) were men, and 0.6% (*n* = 346) were of another gender identity. In terms of race/ethnicity, the majority (74.2%, *n* = 42,269) self-identified as being part of a racialised majority, whereas 11.3% (*n* = 6,448) identified as part of a racialised/ethnic minority group, and 13.5% (*n* = 7,689) were uncertain about their status (race data were not collected in France [*n* = 562; 1.0%] due to a legal prohibition banning the collection and storage of race-based data). In terms of self-reported residence, 27.0% (*n* = 15,408) of participants lived in a capital city, 13.7% (*n* = 7,811) lived in a suburb of a capital city, 25.1% (*n* = 14,319) lived in a provincial city (more than 100,000 residents), 18.7% (*n* = 10,680) lived in a provincial town (more than 10,000 residents), and 15.5% (*n* = 8,750) lived in a rural area. In terms of educational attainment, 0.4% (*n* = 255) reported that they had no formal education, 2.1% (*n* = 1,171) had completed primary education, 17.5% (*n* = 9,954) had completed secondary education, 33.5% (*n* = 19,105) had completed lower tertiary education, 21.5% (*n* = 12,274) had completed higher tertiary education, 21.5% (*n* = 12,262) were in full-time education, and 3.5% (*n* = 1,947) had some other qualification. In terms of marital status, 42.0% (*n* = 23,955) were single, whereas 19.5% (*n* = 11,083) were in a committed relationship but not married, 33.5% (*n* = 19,056) were married, and 5.0% (*n* = 2,874) had another status. With regard to their financial security, 24.9% (*n* = 14,157) of participants reported that they felt less secure relative to others of their own age in their nation of residence, 49.6% (*n* = 28,266) equally secure, and 25.5% (*n* = 14,545) more secure. Table 1 presents detailed sample description data for all individual nations (differentiating between survey presentations in different languages in individual nations).

**Table 1:** Sample descriptions of data from the Body Image in Nature Survey (BINS).

Nation	Sample size	Mean age (*SD*)	%Women	%Ethnic/ racial minority	%Secondary/ tertiary education	%Urban residence	%In committed relationship or married	Mean financial security (*SD*)
Argentina	670	35.36 (13.6)	57	9	81	98	50	2.13 (0.7)
Australia	1,038	35.23 (13.1)	71	18	77	93	55	1.90 (0.8)
Austria	1,279	41.99 (16.5)	54	9	62	67	63	2.08 (0.7)
Bahrain	441	30.47 (9.8)	74	8	87	98	51	1.98 (0.6)
Bangladesh	460	29.30 (8.6)	42	13	80	88	51	1.78 (0.8)
Bosnia & Herzegovina*	406	43.93 (10.9)	64	16	90	87	70	2.15 (0.7)
Brazil	1,462	36.77 (12.0)	58	12	86	99	66	2.21 (0.7)
Bulgaria	248	33.52 (14.1)	62	4	54	92	52	2.16 (0.6)
Canada (English)*	336	24.61 (10.0)	83	14	36	82	48	2.10 (0.7)
Canada (French)	806	38.22 (12.8)	88	7	95	78	72	2.29 (0.7)
Chile	422	36.14 (13.6)	79	8	73	94	41	2.28 (0.8)
China (Cantonese)	409	20.50 (5.9)	58	2	96	100	2	2.18 (0.7)
China (English)	349	21.93 (5.3)	65	6	62	97	26	1.79 (0.7)
China (Mandarin)	1,231	35.00 (7.3)	69	4	92	95	86	1.82 (0.6)
Colombia*	793	27.15 (11.5)	60	7	57	96	22	2.01 (0.8)
Croatia	898	39.10 (12.1)	59	2	91	71	69	2.08 (0.7)
Cyprus	363	34.31 (9.6)	65	4	69	87	64	2.09 (0.7)
Czechia*	700	38.10 (17.0)	66	2	75	82	62	2.29 (0.6)
Ecuador	863	30.97 (12.3)	53	11	65	86	33	1.81 (0.8)
Egypt	1,627	23.62 (8.7)	72	6	86	98	27	2.06 (0.6)
Estonia*	449	38.93 (14.1)	63	2	64	80	58	2.10 (0.7)
France	562	36.01 (14.2)	76	NA	67	64	47	2.08 (0.7)
Germany	620	31.01 (11.9)	62	12	64	83	58	2.18 (0.8)
Ghana	434	21.97 (4.5)	41	26	72	84	32	2.08 (0.8)
Greece*	556	31.49 (11.8)	65	5	63	91	55	2.03 (0.7)
Hungary	654	32.80 (13.4)	69	2	69	72	63	2.07 (0.6)
Iceland (English)	1,149	38.50 (17.5)	50	11	61	92	65	2.27 (0.7)
Iceland (Icelandic)	432	54.91 (15.5)	54	3	81	75	78	2.05 (0.6)
India (Hindi)	1,664	32.07 (11.8)	45	13	78	73	45	2.14 (0.8)
India (Tamil)	376	36.78 (12.1)	52	37	65	57	70	1.71 (0.6)
Indonesia	292	19.79 (3.2)	72	3	43	87	14	1.76 (0.5)
Iran	1,318	33.46 (11.3)	60	29	82	95	61	1.99 (0.6)
Iraq*	405	34.13 (12.1)	33	53	97	100	45	1.49 (0.5)
Ireland	351	33.73 (12.4)	50	5	80	76	62	2.11 (0.8)
Israel	493	30.77 (11.6)	62	7	67	87	32	2.13 (0.7)
Italy	2,307	33.17 (14.0)	62	6	67	81	61	1.95 (0.6)
Japan	360	49.44 (16.6)	100	8	81	90	61	1.79 (0.6)
Kazakhstan	380	30.07 (11.3)	53	11	76	94	48	2.04 (0.6)
Latvia	827	41.04 (12.8)	66	4	82	74	69	2.02 (0.7)
Lebanon	1,295	25.74 (12.3)	67	16	63	70	33	1.93 (0.7)
Lithuania	491	40.34 (12.8)	51	3	84	72	74	2.05 (0.6)
Malaysia	1,193	27.81 (8.7)	69	30	84	76	29	1.74 (0.6)
Malta	347	35.52 (15.4)	72	7	71	78	60	2.10 (0.7)
Nepal	353	25.78 (6.0)	50	5	98	82	28	1.77 (0.7)
Netherlands	1,004	46.81 (16.3)	53	9	98	61	69	2.05 (0.6)
Nigeria	1,274	31.64 (9.2)	34	14	64	93	63	1.85 (0.8)
Norway*	360	41.24 (11.6)	77	4	92	78	77	2.17 (0.7)
Pakistan	267	20.59 (2.7)	28	49	47	100	83	2.16 (0.9)
Palestine*	401	27.64 (9.5)	25	7	90	81	42	2.01 (0.6)
Philippines (English)	350	24.87 (11.2)	0	13	56	97	24	2.03 (0.7)
Philippines (Tagalog)	504	37.43 (11.9)	73	16	89	97	65	1.83 (0.7)
Poland	1,954	30.51 (11.9)	62	3	63	74	56	1.99 (0.7)
Portugal	363	36.53 (17.9)	68	5	81	85	37	2.05 (0.7)
Romania	1,819	26.94 (10.8)	53	5	49	80	60	2.05 (0.7)
Russia*	206	39.94 (11.8)	71	8	84	97	67	1.84 (0.5)
Saudi Arabia*	380	28.02 (9.7)	55	20	83	94	33	2.03 (0.7)
Serbia	650	30.72 (11.3)	56	10	65	95	65	2.20 (0.7)
Slovakia*	814	37.79 (14.7)	54	4	75	65	67	1.92 (0.6)
Slovenia	452	36.84 (14.9)	59	2	87	49	66	2.16 (0.7)
South Africa*	318	35.15 (16.1)	53	31	73	78	45	1.74 (0.8)
South Korea*	381	27.60 (9.7)	48	52	54	98	43	1.89 (0.6)
Spain	1,266	34.54 (16.3)	52	5	82	88	43	2.17 (0.8)
Switzerland	377	46.48 (15.2)	52	5	51	62	66	1.98 (0.7)
Taiwan*	529	41.36 (13.6)	60	7	92	90	67	2.48 (0.7)
Thailand	3,275	25.85 (10.8)	62	6	45	87	23	1.76 (0.6)
Tunisia	374	41.62 (15.2)	55	0	90	96	63	2.10 (0.6)
Türkiye	2,518	31.63 (11.5)	57	14	61	97	57	1.98 (0.8)
Ukraine*	141	39.00 (11.7)	59	9	87	95	71	1.74 (0.6)
United Arab Emirates (Arabic)	204	26.37 (6.7)	73	10	35	99	39	2.07 (0.4)
United Arab Emirates (English)*	904	27.50 (11.8)	36	31	73	98	43	2.13 (0.8)
United Kingdom	1,243	37.99 (13.9)	54	23	87	84	68	2.03 (0.7)
United States of America	2,531	35.35 (12.7)	62	20	82	85	61	1.93 (0.7)

*Note*. * National groups utilising a 5-point response scale in the Satisfaction With Life Scale. *SD* = standard deviation.

### Measures

#### Life satisfaction

As part of the BINS survey package, participants completed the 5-item Satisfaction with Life Scale (SWLS) [11], which has previously been shown to have adequate composite reliability and evidence of nomological validity in diverse cultural contexts [3]. In most participating nations, participants completed the SWLS using a 7-point response scale (1 = *strongly disagree*, 7 = *strongly agree*). However, due to administrative oversights, a 5-point response scale (1 = *strongly disagree*, 5 = *strongly agree*) was used instead in Bosnia and Herzegovina, Canada (when the survey was presented in English, but not in French), Colombia, Czechia, Estonia, Greece, Iraq, Norway, Palestine, Russia, Saudi Arabia, Slovakia, South Africa, South Korea, Taiwan, Ukraine, and the United Arab Emirates (when the survey was presented in English, but not in Arabic). Unless presented in English, or where a previously validated translation was not available, the SWLS was translated for use in BINS using the parallel back-translation procedure [93] (for further information, see [90]). A list of the 40 languages in which the BINS survey package was presented is reported in S1 Table.

#### Financial security

Following previous cross-national work [94,95], participants were asked to self-report how financially secure they felt relative to others of their own age in their country of residence (1 = *less secure*, 2 = *same*, 3 = *more secure*).

#### Urbanicity

To assess urbanicity, participants were asked about their current place of residence, with response options adapted from Pedersen and Mortensen [96] as follows: *capital city*, *capital city suburbs*, *provincial city (more than 100*,*000 residents)*, *provincial town (more than 10*,*000 residents)*, and *rural areas*. Response options were assigned values 1 to 5 (in the above order) for statistical analysis and collapsed into *urban* versus *rural* for descriptive purposes. This measure of urbanicity has been used in previous cross-national work [95].

#### Demographics

Participants were asked to provide their demographic data consisting of gender identity (1 = *woman*, 2 = *man*, 3 = *describe gender in another way*), age (open-ended), highest educational qualification (1 = *no formal education*, 2 = *primary education*, 3 = *secondary education*, 4 = *still in full-time education*, 5 = *undergraduate degree*, 6 = *postgraduate degree*, 7 = *other*), marital status (1 = *single*, 2 = *single but in a committed relationship*, 3 = *married*, 4 = *other*), and ethnicity/race (1 = *ethnic/racial majority*, 2 = *ethnic/racial minority*, 3 = *not sure*). The latter item was included as it provides a common metric of categorising ethnicity/race across diverse nations [95]. For descriptive purposes at the national level and for analyses, response options for highest educational qualification were collapsed into *secondary/tertiary* (secondary education, undergraduate degree, postgraduate degree) versus *other* (all remaining categories) and response options of marital status were collapsed into *committed/married* (single but in a committed relationship, married) versus *other* (all remaining categories). Response options of ethnicity/race were collapsed into *racialised minority* (racial minority) versus *other* (all remaining categories).

### Procedures, ethics, and data sharing

Full procedural information about the BINS is provided elsewhere [90]. The BINS project was conducted in accordance with the principles of the Declaration of Helsinki [97] and following all local institutional guidelines. In brief, once local ethics approval had been obtained or collaborators confirmed that approval was not required as per national laws (see S1 Table), researchers recruited participants from the community in their respective nations between November 2020 and February 2022. Inclusion criteria were being ≥ 18 years of age, a resident and citizen of the particular nation in which recruitment took place, and being able to complete a survey in the language in which it was presented. In all but nine locales (S1 Table), data collection was conducted online. All participants were presented with a standardised information sheet and provided (digital or written) informed consent before completing an anonymous version of the BINS survey package. Upon completion of the survey, participants received debriefing information, which included contact information for the first author as well as a local researcher. The BINS data, codebook, and our analytic codes are available on the Open Science Framework at https://osf.io/kzq7w/.

### Analytic strategy

The general analytic plan for the structural and measurement invariance analyses of the key variables of the BINS, including the SWLS, is outlined in the BINS study protocol [90]. Unless mentioned below, Mplus 8.8 [98] was used for all analyses, using full-information maximum likelihood estimation to account for partially missing data. Measurement invariance was assessed through the use of multi-group confirmatory factor analysis (MG-CFA) [29], testing for configural, metric, and scalar invariance, in this sequence; that is, whether all five SWLS items loaded on a single underlying factor in all groups (configural invariance), whether item loadings were the same in all groups (metric invariance), and whether item intercepts were the same in all groups (scalar invariance). Following previous recommendations [41,72], all models included a residual covariance between Items #4 and #5 (i.e., the two SWLS items referring to past life satisfaction).

Groups were constituted in four separate analyses: nations, languages, gender identities (women vs. men vs. other gender identities), and age (18–24 years vs. 25–44 years vs. ≥ 45 years). The analysis of nations was listed in the BINS study protocol for all key variables [90], whereas analyses of languages, gender identities, and age groups applied specifically to the SWLS and were not mentioned in the more general study protocol. Prior to testing measurement invariance across nations, invariance of the cross-language survey presentation (i.e., where surveys were presented in more than one language in a single nation) in Canada, China, Iceland, India, the Philippines, and the United Arab Emirates was tested. Data from these nations were also entered in invariance tests across nations twice (thrice in the case of China): once with (one of) their native language(s) (two native languages in the case of China) and a second time with English (Tamil in India). For conceptual clarity, we refer to “national groups” instead of “nations” in the first set of analyses.

Item parameters were relaxed if measurement invariance did not hold, thereby aiming to achieve partial measurement invariance (i.e., equal item parameters across some groups and items, but not all). However, because of the large number of groups in the analyses of national groups and languages, the alignment method was applied here for guidance. The alignment method [99,100] does not require exact measurement invariance, but is based on the notion of approximate measurement invariance. It begins with the configural invariance model and seeks a solution that minimises the differences in loadings and intercepts across groups, while still retaining identical fit to the configural invariance model. Alignment provides quantitative information on the amount of deviation from scalar measurement invariance for the overall set of items and on the groups and items for which measurement invariance concerning either loadings and/or intercepts holds. While alignment also provides adjusted latent means and variances in line with the optimal alignment of loadings and intercepts across groups, these estimates are expected to lose validity when a larger number of item parameters is non-invariant (e.g., > 20%) [99,100]. Thus, we utilised the alignment method to identify national groups that possibly needed to be excluded to achieve acceptable model fit and to identify items that could be used as anchor items (two items as a minimum for the estimation of latent means; [101]; for a recent simulation study confirming the accuracy of such an approach, see [102]) in partial scalar measurement invariance models. We present information on latent mean differences between groups in all analyses based on either the full or partial scalar MG-CFA models, where applicable. For comparison, we also report on the agreement (intraclass correlation [ICC]; two-way mixed model, absolute agreement definition) [103] of rankings of latent means as provided by the partial measurement models and the alignment method, where applicable. We also present reliability estimates (ω total) [104] for the SWLS, based on the configural invariance models in groups in all analyses.

Sociodemographic correlates of life satisfaction were examined with a multilevel model across the national groups. The factor scores of national groups were used as dependent variables. The groupmean-centred variables of financial security, urbanicity, education, marital status, and racialised status were used as Level-1 predictors, and the cluster-level means of these variables were used as Level-2 predictors. Thereby, this model optimally distinguished Level-1 from Level-2 effects and investigated associations of the predictors with the outcome both at the individual level (Level 1) within national groups, but also at the cluster level (Level 2) between national groups. We also included age as a control variable (both on Level 1 and 2), as there was some indication of age differences in life satisfaction in the present data (see Results). To prevent overfitting on Level 2 (where the ratio of predictors to groups was relatively high), only significant Level-2 predictors (*p* < .05, two-tailed) were retained in the final model. Bayesian estimation (using diffuse priors as specified in Mplus default settings) was used to obtain parameter estimates on a standardised scale, which were interpreted as measures of effect size (similar to Pearson’s *r*). As the interpretation of standardised estimates of multilevel models can be misleading in certain situations [105], we also provide estimates of effect size in the metric of *R*^2^ for both the overall model and each individual predictor. The *R* package *r2mlm* [106] was used for these computations. We report *R*^2^ values for the proportions of within-cluster outcome variance attributable to the Level-1 predictors via fixed slopes and the between-cluster outcome variance attributable to the Level-2 predictors via fixed slopes, respectively.

There were 190 missing values in total (0.07%) in the items of the SWLS. As the SWLS was presented with both 5-point and 7-point response scales, we opted to use in the structural analyses the robust maximum likelihood estimator (MLR) instead of the mean- and variance-adjusted weighted least squares estimator (WLSMV), as was stated in the study protocol [90]. WLSMV estimation is specifically suited to ordered-categorical item response formats but requires the same number of item response options across all groups in a multi-group context. MLR is an alternative to WLSMV [107] and can deal with the different numbers of response options in the current data. MLR estimates one intercept parameter per item, irrespective of the number of response options, whereas WLSMV estimates threshold parameters for each pair of response options for each item. Prior to analyses, the 5-point scales were numerically equated to the 7-point response scales by applying the scale transformation formulas described by Aiken [108], replacing the original scale values 1, 2, 3, 4, and 5 with the equated scale values of 1.2, 2.6, 4, 5.4, and 6.8, respectively.

For the assessment of model fit, the comparative fit index (CFI) and the Tucker-Lewis index (TLI; values close to .95 indicative of good fit), the root-mean square error of approximation (RMSEA) and its 90% confidence interval (values close to .06 indicative of good fit), and the standardised root mean square residual (SRMR; values close to .08 indicative of good fit) [109] are reported. For MG-CFAs with more than 10 groups, we used a higher cut-off for the RMSEA of .15, as RMSEA values tend to be inflated with increasing number of groups [110]. For the comparison of configural, metric, and scalar invariance models in the MG-CFAs, ΔCFI and ΔRMSEA values, and Δχ^2^ tests are presented. We primarily interpreted ΔCFI and ΔRMSEA values, which were not affected by the large sample size of the current study, but also consulted the overall fit of these models for their comparative evaluation. Cut-offs recommended by Rutkowski and Svetina [110] were utilised, with ΔCFI ≲ .020 and ΔRMSEA ≲ .030 taken as indication of good fit of metric invariance models in comparison to configural invariance models, and ΔCFI ≲ .010 and ΔRMSEA ≲ .015 as indication of good fit of scalar invariance models in comparison to metric invariance models.

## Results

### Invariance of cross-language survey presentation in six countries

Results of the MG-CFAs testing the invariance of the cross-language survey presentation in Canada, China, Iceland, India, the Philippines, and the United Arab Emirates (UAE) are presented in S2 Table. We obtained evidence of configural invariance of the SWLS across languages for all of these countries. Judging from the overall fit and ΔCFI values, metric invariance could be assumed for China, Iceland, and India, and the UAE (but note that the UAE Arabic sample was rather small, *n*s = 204 vs. 904 for the UAE English sample; thus, the invariance test for the UAE data likely had low power). However, scalar invariance was not achieved for any of the countries, though scalar invariance was almost achieved in the languages presented in Canada, China, and India (see S2 Table).

### Invariance concerning national group, language, gender identities, and age

#### Overall findings

Table 2 presents the results of the MG-CFAs. Across all 72 national groups and 40 languages, the SWLS demonstrated configural and metric invariance (based on the ΔRMSEA values and sufficiently close based on the ΔCFI value for the national groups), but not scalar invariance. Across the three gender identity groups and three age groups, the SWLS demonstrated scalar invariance (scalar invariance also held for national groups with 5- and 7-point response options in a supplementary analysis; see S2 Table). Standardised loadings and intercepts of the SWLS items in the gender identity and age groups are presented in Table 3. Gender identities differed in latent means by Cohen’s *d* = 0.004 (*p* = .63; women vs. men) and *d* = -0.59 (*p* < .001; women vs. other gender identities); age groups by *d* = 0.06 (*p* < .001; 18–24 years vs. 25–44 years) and *d* = 0.13 (*p* < .001; 18–24 years vs. ≥ 45 years). Scale reliabilities (ω total) ranged from .47 (United Arab Emirates [Arabic]) to .93 (Japan) across the national groups (see S1 Fig for a histogram), with a median of .85 (P_25_ = .83, P_75_ = .87), and from .63 (Tamil) to .93 (Japanese) across languages, with a median of .86 (P_25_ = .83, P_75_ = .87). Scale reliabilities were .86, .84, and .85 for women, men, and other gender identities, and .83, .86, and .88 for the age groups of 18–24 years, 25–44 years, and ≥ 45 years.

**Table 2:** Invariance concerning national groups, language, gender identity, and age.

							Model comparisons			
Grouping variable	χ^2^(*df*)	CFI	TLI	RMSEA	95% *CI*	SRMR	ΔCFI	ΔRMSEA	Configural	Metric
National Groups										
Configural invariance	1708.14(288)	.983	.957	.079	[.075, .083]	.027				
Metric invariance	3792.94(572)	.961	.951	.084	[.082, .087]	.091	.022	.005	2147.15(284)	
Scalar invariance	12964.75(856)	.853	.876	.134	[.132, .136]	.133	.108	.050	12271.01(568)	10594.63(284)
Language										
Configural invariance	1223.53(160)	.987	.966	.068	[.065, .072]	.017				
Metric invariance	2545.41(316)	.972	.964	.070	[.068, .073]	.066	.015	.002	1342.29(156)	
Scalar invariance	9627.52(472)	.885	.902	.117	[.115, .119]	.104	.087	.047	9209.21(312)	8302.98(156)
Gender identity										
Configural invariance	335.42(12)	.996	.989	.038	[.034, .041]	.009				
Metric invariance	427.60(20)	.994	.992	.033	[.030, .036]	.012	.002	-.005	24.71(8)^a^	
Scalar invariance	654.57(28)	.991	.991	.034	[.032, .037]	.017	.003	.001	271.83(16)	247.91(8)
Age										
Configural invariance	410.93(12)	.994	.986	.042	[.038, .045]	.010				
Metric invariance	500.75(20)	.993	.990	.036	[.033, .038]	.012	.001	-.006	21.92(8)^b^	
Scalar invariance	1119.44(28)	.985	.983	.045	[.043, .048]	.021	.008	.009	755.92(16)	768.35(8)

*Note*. All *p*s of χ^2^ and Δχ^2^ tests (model comparisons, against a configural invariance model [Configural column] or a metric invariance model [Metric column]) were < .001, except where noted otherwise. Gender identity compared groups of women, men, and other gender identity, age compared groups of participants with 18–24 years, 25–44 years, ≥ 45 years of age.

^a^ *p* = .002.

^b^ *p* = .005.

**Table 3:** Standardised SWLS item loadings and intercepts in the gender identity and age groups.

Parameter and item	Gender identity (women/men/other)	Age (18–24 years/25-44 years/≥ 45 years)
Loadings		
Item #1	.85/.84/.80	.80/.86/.89
Item #2	.78/.76/.75	.75/.78/.80
Item #3	.81/.80/.77	.78/.82/.84
Item #4	.74/.72/.71	.69/.74/.79
Item #5	.63/.61/.61	.59/.63/.66
Covariance Items #4 & #5	.15/.18/.09^a^	.16/.17/.14
Intercepts		
Item #1	2.86/2.79/2.70	2.75/2.81/2.81
Item #2	2.99/2.96/2.79	2.88/2.96/2.96
Item #3	3.10/3.06/2.87	2.94/3.08/3.10
Item #4	2.86/2.77/2.67	2.68/2.82/2.92
Item #5	2.13/2.08/2.04	2.01/2.11/2.14

*Note*. Unstandardised parameters were restricted to equality in the scalar measurement invariance MG-CFA models that were used to estimate the parameters; standardised estimates may still differ from one group to another due to differences in dispersion. All *p*s < .001, except where noted otherwise. ^a^ *p* = .20.

#### National group

Using the alignment method, optimal item loadings and intercepts were obtained for the national groups (see S3 Table). The items of the SWLS demonstrated invariance of loadings across 55 (Item #2) to 69 (Item #4) of the 72 national groups (including six nations with more than one survey language) and invariance of intercepts across 38 (Item #1) to 47 (Item #3) national groups. The two most invariant items across both loading and intercept parameters were Items #1 and #3, considering the respective numbers of national groups showing invariance, but also taking into account individual *R*^2^ values of the item parameters (see S3 Table). This index can be interpreted as the extent to which the respective SWLS item parameter was invariant (0 = no invariance, 1 = full invariance).

However, there are a number of specific reasons why *R*^2^ values could be low even though invariance is high [86]. Thus, even though *R*^2^ of the loading parameter of Item #3 was apparently 0 (see S3 Table), it was invariant in 63 of the 72 national groups (see S2 Fig for a heatmap). Furthermore, of all five items, the intercept parameter of Item #3 had the second highest *R*^2^ value and was invariant in the largest number of national groups. All five items had invariant loadings (metric invariance) across 37 national groups and invariant intercepts across 11 national groups. Scalar invariance (invariant loadings and intercepts) of all five items held across seven national groups: Canada (English), Cyprus, Lithuania, the Philippines (English), Serbia, and Slovenia, and the United Arab Emirates (English). Across all national groups and item parameters, the average invariance index for the SWLS was .515.

We used the information provided by MG-CFAs and the alignment method to construct a partial scalar measurement invariance model that allowed us to compare national groups on the latent mean level. First, data from Iraq and Nigeria were excluded as these countries each had large contributions, relative to their sample sizes, to all three χ^2^ tests of the MG-CFA invariance models. Second, data from the United Arab Emirates (Arabic) was excluded, as scale reliability was unacceptably low there (see above). Refitting the metric invariance MG-CFA model to the reduced set of national groups resulted in a fit that allowed accepting metric invariance, χ^2^ = 2745.74, *df* = 548, *p* < .001, CFI = .973, TLI = .966, RMSEA = .071, 90% CI = [.068, .074], SRMR = .071. This model also compared favourably to the configural invariance model of the reduced set, χ^2^ = 1191.32, *df* = 276, *p* < .001, CFI = .989, TLI = .972, RMSEA = .064, 90% CI = [.061, .068], SRMR = .017, with comparative test and indices of Δχ^2^ = 1614.05, *df* = 272, *p* < .001, ΔCFI = .016, ΔRMSEA = .005.

Third, Items #1 and #3 were selected as anchor items; that is, the partial scalar measurement model assumed scalar invariance only for these two items; all parameters of the other items were estimated freely. The partial scalar measurement model had a good fit to the data, χ^2^ = 3699.59, *df* = 412, *p* < .001, CFI = .959, TLI = .932, RMSEA = .100, 90% CI = [.097, .103], SRMR = .056, and also compared reasonably well to the above metric invariance model^2^, ΔCFI = .014, ΔRMSEA = .029.

**Fig 1:**
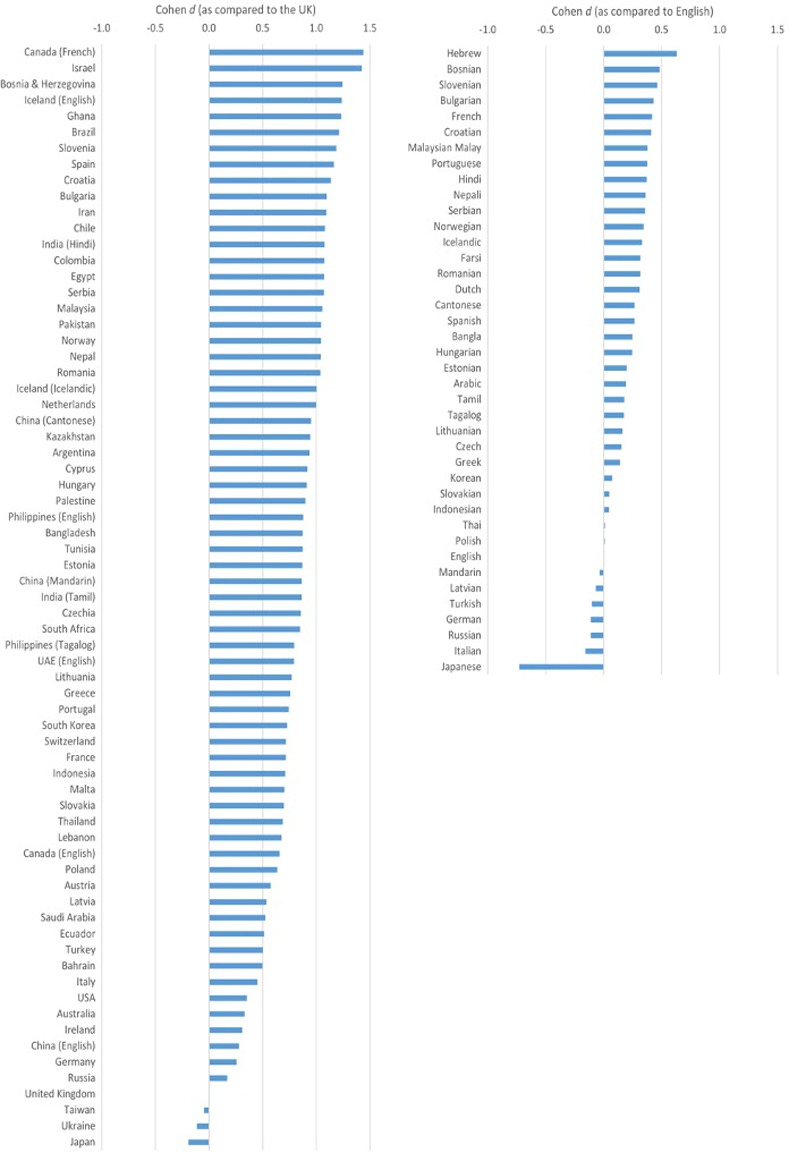
Ordering and magnitude of standardised latent mean differences (Cohen’s d) between national groups (as compared to the United Kingdom; Left) and languages (as compared to English; Right).

The ordering and magnitude of standardised latent mean differences (Cohen’s *d*) between national groups (as compared to the United Kingdom, which served as an anchor in this analysis) are provided in Fig 1 (individual Cohen’s *d* values are provided in S4 Table). The largest positive differences between national groups, as compared to the United Kingdom, were observed for (in descending order) Canada (French), Israel, and Bosnia and Herzegovina and were in the range of *d* = 1.25 to 1.44 (see Fig 1, left), suggesting that participants from these countries reported the highest life satisfaction. The largest negative differences were observed for Taiwan, Japan, and Ukraine and were in the range of *d* = -0.05 to -0.19. The rankings of national groups according to their latent means, as provided by the partial measurement model and the alignment method (obtained with the reduced set of national groups), agreed at ICC = .92 (see S3 Fig for a scatterplot).

#### Languages

Using the alignment method, optimal item loadings and intercepts were obtained for languages (see S5 Table). The items of the SWLS demonstrated invariance of loadings across 31 (Item #5) to 38 (Item #1) of the 40 languages and invariance of intercepts across 19 (Items #2 and #3) to 25 (Item #5) languages. Items #1 and #4 appeared to be the two most invariant items concerning the loading parameters (closely followed by Item #3); with respect to the intercept parameters, Item #5 appeared to be the most invariant concerning the number of languages, but Items #2 and #3 concerning their *R*^2^ value. All five items had invariant loadings (metric invariance) across 17 languages and invariant intercepts in three languages. Scalar invariance (invariant loadings and intercepts) of all five items held only across Bulgarian but none of the investigated languages. Across all languages and item parameters, the average invariance index for the SWLS was .613, which was slightly higher than for the national groups.

As the metric invariance model showed acceptable fit (see Table 2), no languages were excluded from further analysis. Using Items #1 and #3 as anchor items, the partial scalar measurement model had a good fit to the data, χ^2^ = 3433.80, *df* = 238, *p* < .001, CFI = .960, TLI = .932, RMSEA = .097, 90% CI = [.094, .100], SRMR = .050, and also compared reasonably well to the metric invariance model, ΔCFI = .012, ΔRMSEA = .027.

The ordering and magnitude of standardised latent mean differences (Cohen’s *d*) between languages (as compared to English, which served as an anchor here) are provided in Fig 1 (individual Cohen’s *d* values are provided in S6 Table). The largest positive differences between languages, as compared to English, were observed for Hebrew, Bosnian, and Slovenian and were in the range of *d* = 0.46 to 0.63 (Fig 1, right). The largest negative differences were observed for Russian, Italian, and Japanese and were in the range of *d* = -0.11 to -0.73. The rankings of languages according to their latent means, as provided by the partial measurement model and the alignment method, agreed at ICC = .88 (see S3 Fig for a scatterplot).

### Sociodemographic correlates of life satisfaction

The results of the final multilevel model are presented in Table 4. Only the cluster-level means of financial security were kept as a Level-2 predictor in this model. Higher financial security, secondary or tertiary education (vs. other), being in a committed relationship or married (vs. other), older age, and identifying as part of a racialised majority (vs. other) were associated with higher life satisfaction at the individual level. Satisfaction with life was further higher in national groups with higher cluster-level means of financial security. Restating the effects of the dichotomous predictors at the individual level in the metric of Cohen’s *d*, the effects of education, marital status, and racialised status amounted to *d* = 0.06, 0.22, and -0.05, respectively. According to the magnitude of both the standardised parameter estimates and the corresponding *R*^2^ values, financial security was the single most important predictor for life satisfaction (on both Levels 1 and 2; explaining roughly 10% of the outcome variance on each level), followed by marital status (1% explained variance on Level 1). All other predictors explained only negligible proportions of life satisfaction.

**Table 4:** Results of the multilevel analysis on the correlates of life satisfaction.

Predictor	Estimate (posterior *SD*)	95% credibility interval	*p* (one-tailed)	Δ*R*^2^
Level 1				
Financial security	0.30 (0.004)	[0.29, 0.31]	< .001	9%
Urbanicity	0.002 (0.004)	[-0.006, 0.01]	.19	0%
Education	0.03 (0.005)	[0.02, 0.04]	< .001	0%
Marital status	0.11 (0.004)	[0.10, 0.12]	< .001	1%
Racialised status	-0.02 (0.004)	[-0.03, -0.01]	< .001	0%
Age	0.01 (0.005)	[0.001, 0.02]	< .001	0%
Level 2: cluster-level means				
Financial security	0.33 (0.11)	[0.11, 0.50]	< .001	10%
*Random Effects*				
Intercept	0.89 (0.07)	[0.75, 0.99]	< .001	
*R*^2^ *(Level 1/Level 2)*	11% / 10%			

*Note*. Estimates are on a standardised scale. *R*^2^ (Level 1/Level 2) quantifies the proportions of within-cluster outcome variance attributable to the Level-1 predictors via fixed slopes and the between-cluster outcome variance attributable to the Level-2 predictors via fixed slopes, respectively. Δ*R*^2^ quantifies the contributions of each predictor to these proportions (on Level 1 for Level-1 predictors, and on Level 2 for Level-2 predictors), by comparing the full model with a model without this predictor. In the case of financial security, the predictor was removed from Level 1 and Level 2 in tandem. The analysis did not include data from France, as minority status was not assessed there. However, parameter estimates did not differ when minority status was excluded as a predictor from the model, and data from France were included in analysis. *SD* = standard deviation.

## Discussion

In the present study, we used the BINS dataset, consisting of data from respondents in 65 nations, to assess measurement invariance of the SWLS. In this dataset, there was clear evidence of configural invariance across national groups, languages, gender identities, and age groups. These results suggest that the unidimensional model of SWLS scores and its pattern of loadings is the same across all four categories that were examined here; that is, all five items of the SWLS load onto a single life satisfaction factor irrespective of respondent characteristics (i.e., national group, gender identities, and age group) or survey presentation (i.e., survey language). Beyond this, the results of the present study also evidenced full scalar invariance across gender identities and age groups, whereas partial scalar invariance was supported across almost all of the national groups and across all languages represented in the BINS. These findings are consistent with previous large-scale cross-national studies (where 24–26 nations have been assessed; [40,41]), but also add uniquely to the literature in a number of ways, as we elaborate upon below.

### Measurement invariance across national groups

In terms of national groups represented in the BINS dataset, our results indicated support for full metric invariance for the unidimensional model with correlated residuals between Items #4 and 5 (i.e., the two items that tap past life satisfaction). Indeed, the individual items of the SWLS showed invariance of loadings across a near majority of all national groups (i.e., including six nations with more than one survey language). These results suggest that each SWLS item contributes to the manifest variable reflecting overall life satisfaction to a similar degree across the majority of national groups that were represented in the BINS, which in turn means that the SWLS could be used to explain differences in correlates of life satisfaction across nations [29,30]. Items #1 (“In most ways, my life is close to my ideal”) and #3 (“I am satisfied with my life”), in particular showed the highest degree of metric invariance, which is similar to the findings of Jang and colleagues [40].

Beyond metric invariance, our data supported full scalar invariance across a small number of the national groups represented in the BINS (seven of the 72 national groups). However, once we excluded data from three countries (Nigeria, Iraq, and the United Arab Emirates) that contributed substantially to metric non-invariance and once we selected Items #1 and #3 as anchor items (i.e., partial scalar invariance was assumed for these items, with all parameters of other items freely estimated), our data indicated evidence of partial scalar invariance. Inspection of parameters across national groups suggested that the least invariant item was Item #2 (“The conditions of my life are excellent”). This item appears to also have been problematic in terms of scalar invariance in previous cross-national studies [33,40]. One possible explanation for this finding is that the meaning of the term “excellent” has different denotations or connotations across national groups. For instance, it is possible that some national groups believe that one can be satisfied with life without all conditions of life being “excellent” [48] or that “excellent” conditions of life are not uniformly desirable across nations [111]. If this is the case, then this item may contribute differentially to overall life satisfaction across nations.

When considering scalar invariance, it is worth bearing in mind full scalar invariance is often an unrealistic goal for datasets with a larger number of groups [112]. As such, the evidence of partial scalar invariance presented here can be viewed as noteworthy, especially given the very large number of national groups represented in our analyses. Moreover, the present results are broadly consistent with the findings of Jang and colleagues [40]–particularly in terms of the scalar invariance of Items #1 and #3 –and suggest that it is possible to compare latent SWLS means across national groups with some caution. Based on rank-factoring of latent means, the BINS dataset indicated wide variability in overall life satisfaction in the context of the COVID-19 pandemic, with respondents in (French-speaking) Canada and Israel having the highest SWLS means, and respondents in Japan and Ukraine having the lowest means. In considering these findings, however, it is worth remembering that even a small number of unequal indicator intercepts can substantially affect latent means [113]. While caution is needed when interpreting the latent means presented in Fig 1, one broad conclusion might be that life satisfaction varied substantially across national groups at the time of the COVID-19 pandemic. Nevertheless, practitioners and policy-makers may find these data useful when attempting to understand differences in life satisfaction between nations, as well as how to better support subjective well-being in the post-pandemic era.

### Measurement invariance across languages

Notably, very few previous studies have assessed the measurement invariance of the SWLS across languages. Here, and similar to our findings *vis-à-vis* national groups, we found that the SWLS achieved full metric but not full scalar invariance. Although the average invariance index for the SWLS was slightly higher across languages than it was across national groups, it was notable that scalar invariance across all five SWLS items did not hold across any of the languages investigated in the BINS. Nevertheless, when Items #1 and #3 were selected as anchors with parameters for all other items freely estimated, the current results presented evidence of partial scalar invariance across the 40 languages represented in the BINS. To our knowledge, this is the first demonstration that the SWLS achieves partial scalar invariance across a large number of languages but also suggests that linguistic structures (e.g., grammar, vocabulary) and understandings (e.g., cultural or linguistic differences in meaning) may have an impact on the way in which the SWLS items are understood.

One broad conclusion here is that it may not be possible to translate certain concepts represented in the SWLS in perfectly equivalent ways across languages, which in turn likely means that something is lost and/or something is added when the SWLS is translated into new languages [114]. As an example, Lolle and Anderson [45] have suggested that the way in which scale anchors are translated and understood by respondents from different linguistic backgrounds may affect how life satisfaction instruments are completed (e.g., “strongly agree” could be translated to mean “very much agree” or “highly agree”, which could have different connotations). Likewise, it is possible that differences in the way in which specific concepts represented in the SWLS are translated could have affected metric and scalar invariance. To take Item #2 (the SWLS item that was most problematic in terms of invariance of intercepts), it is possible that understandings and connotations of “excellent” vary across languages. Likewise, it is also possible that linguistic structures affected what is denoted by “conditions of my life” in Item #2 (e.g., whether one relies on affective or sociocultural understandings of the term) [46]. Indeed, even small differences in translatory meaning could have resulted in large impacts on scalar invariance [60].

Nevertheless, on the basis of partial scalar invariance, we found that respondents completing the SWLS in Hebrew ranked highest in terms of latent means, whereas respondents completing the SWLS in Japanese had the lowest means (which likely reflects national group differences discussed above). Interestingly, the BINS dataset also provided an opportunity to examine measurement invariance of the SWLS across languages *within* six nations (i.e., where the SWLS was presented in more than one language in a single nation). Full metric invariance was achieved across four of the six countries, but full scalar invariance was problematic in all six countries. These findings suggest that the language in which the SWLS is presented within particular nations may affect how it is understood and completed, which in turn has important implications for scholars and practitioners when deciding how best to present the instrument. Indeed, our results can be taken as support for the conclusions of Lolle and Anderson [45], who suggested that linguistic differences may sometimes result in a “semantic loss and gain” (p. 1339) that impacts on measurement invariance of life satisfaction instruments.

### Measurement invariance across gender identities and age

The results with regard to measurement invariance across national groups and languages suggested that understandings of life satisfaction are complex and that responses to even seemingly simple instruments like the SWLS may be affected by one’s national group and linguistic background. In contrast, the present analyses supported full scalar invariance of the SWLS across gender identities and age groups. In terms of gender identity, our results are consistent with previous cross-national work showing that the SWLS typically achieves at least partial scalar invariance [38,41]. Put differently, it is unlikely that one’s gendered experiences affect the way in which the construct of life satisfaction, as measured using the SWLS, is understood [37]. Moreover, and consistent with previous work [14,115], latent mean differences between women and men on the SWLS were negligible and not significant. To our knowledge, however, this is the first study to examine measurement invariance of the SWLS beyond the binary of women versus men, with our results suggesting that those who identified as another gender had significantly lower SWLS scores than women and men. Although this finding is interesting and consistent with other work showing that those who described their gender in another way have poorer life satisfaction [116], possibly because of gender minority stress [117], it should also be remembered that respondents who identified as another gender were relatively small in number (0.6% of the total dataset).

Our findings are also consistent with the few studies that have assessed age invariance, suggesting that the SWLS is scalar invariant across age groups in adulthood. For instance, in samples of Spanish adults [118] and German adults [119], the SWLS has been shown to be invariant across age groups. Although our age groupings were different to these aforementioned studies (i.e., we grouped participants according to commonly used stages of adulthood) [91,92], one broad conclusion is that understandings and meanings of life satisfaction and subjective well-being are relatively stable throughout adulthood [120]. However, in contrast to these aforementioned studies, where no linear trends in latent means were observed [121], our results suggest that SWLS scores were significantly higher with older age. Still, comparisons of latent means across age groups suggested that significant differences were negligible-to-small in size, suggesting that life satisfaction remains relatively stable in adulthood, as has been reported elsewhere [122,123]. It should be remembered, however, that our dataset was limited to adults, and there is some evidence that adolescents may have significantly greater life satisfaction than adults [124].

### Correlates of life satisfaction

The BINS dataset also allowed us to examine associations between life satisfaction and key sociodemographic variables at the level of the individual. Our results indicated that greater life satisfaction was significantly associated with greater financial security higher educational qualifications (which may be a proxy of socioeconomic status, although the effect size of this particular relationship was negligible). Overall, these findings are consistent with the wealth of evidence indicating that a fulfilment of basic human needs and greater material well-being is a strong–possibly the strongest [73]–predictor of psychological well-being generally, and life satisfaction specifically [7,74–76]. Although conceptions of material well-being may well differ across cultural contexts [89], our results suggest that, when considered at the level of the individual, the association between life satisfaction and proxies of material well-being appear to be relatively robust across nations. These results are important because they suggest that, across nations, improving material well-being may be one direct way of promoting greater life satisfaction.

Additionally, we found that marital status was significantly associated with life satisfaction. More specifically, we found that respondents in committed relationships were more likely to report greater life satisfaction than those who were unpartnered. This finding is consistent with the broader literature showing that marital status is a robust correlate of psychological well-being [77]. In explanation, it has been suggested that the formation and maintenance of social relationships is central to the promotion of life satisfaction [78,125], particularly in cultures or communities that emphasise family values [126], because they satisfy a basic human need for belongingness and are a source of positive affirmation [127], reinforce healthy behaviours, and buffer against the impact of negative life events [128]. These issues would likely have been amplified during the COVID-19 pandemic, such that those in committed relationships were able to rely on their partners despite periods of social isolation [129]. Of course, we recognise that focusing on relationship status does not allow us to elucidate the underlying mechanisms through which relationship status affects life satisfaction. It seems likely, however, that those in committed relationships may be able to report greater emotional support and social integration, which in turn contributes independently to life satisfaction [86].

In contrast, we found that identification as a part of a racialised minority was associated with lower life satisfaction. Although this is consistent with some previous work within nations [86–88] and likely reflects the deleterious effect of experiences of racism on life satisfaction [130,131], it should be noted that the effect size of this relationship was negligible. Finally, we found that urbanicity was only negligibly associated with life satisfaction. As we noted earlier, studies examining associations between urbanicity and life satisfaction have returned equivocal results [79,81–85]. In this regard, our results are consistent with recent work suggesting that the effects of urbanicity on life satisfaction are negligible at best [132]. Overall, then, our results suggest that financial security and marital status were more robustly associated with life satisfaction across nations, whereas other correlates–including racialised identity and urbanicity–showed only negligible associations.

### Constraints on generalisability

A number of constraints on the generalisability [133] of the present findings should be considered. Most importantly, although the BINS dataset presents a unique opportunity to examine measurement invariance of the SWLS, our findings may be limited in terms of their generalisability because of sampling and recruitment constraints. For instance, across research sites, we recruited participants opportunistically and, as such, our subsamples cannot be considered representative of their respective nations. We also did not keep records of response rates, which means that we are unable to estimate the effects of non-response bias. In addition, racialised identity in the present study was measured using a relatively blunt tool (i.e., we asked whether participants identified as part of an ethnic/racial majority or minority [95]), which may mean that varying constructs were conflated (e.g., physical characteristics, common ancestry, cultural identification, etc.). Moreover, although our inclusion criteria included being a resident and citizen of the nation in which recruitment took place, we did not ask about migration status (e.g., whether participants were born and raised in their respective nations), which may have added a degree of lack of representativeness. In a similar vein, although attempts were made to ensure operational equivalence [90], small differences in recruitment and survey completion cannot entirely be ruled out (most notably differences between online and paper-and-pencil survey formats). In practice, of course, ensuring complete operational equivalence across a diverse and large range of nations and cultural contexts may be very difficult. Moreover, even if our samples do not guarantee representativeness, they nevertheless provide very good snapshots of national populations given our largely consistent recruitment methods across study sites.

Likewise, although the BINS dataset provides what is currently the widest representation of SWLS scores across nations, it should be noted that the BINS was under-represented in a number of world regions, particularly Africa, Central Asia, the Caribbean, and Central America. Similarly, although the BINS dataset provides a very useful portrait of life satisfaction in the shadow of the COVID-19 pandemic, specific conditions during the period of data collection may have shaped our findings. Indeed, the extended time horizon of data collection (i.e., over about two years) may have introduced nation-specific biases, especially as experiences and manifestations of psychological well-being likely fluctuated during the pandemic [134] and may have been affected by such factors as being in lockdown, the severity of the pandemic locally, and intra- and inter-national responses to the pandemic (which were not assessed in our survey). We also cannot entirely rule out common method biases, as the BINS dataset consists of self-reported data. Nevertheless, we adopted a raft of *ex ante* strategies to minimise common method variance, including assuring respondents of their anonymity and confidentiality, ensuring that items in the BINS survey were formulated concisely, and utilising different scale endpoints [135]. Having said that, because the instruments included in the BINS survey were not randomised during presentation, responses to the SWLS may have been affected by its position within the broader survey–though any impact is likely to have been marginal [136]. Finally, in future work, it may also be useful to examine the impact of additional factors that were not measured in the BINS, such as religious identity [137,138] and socio-political capital (e.g., political stability, corruption) [139], as these may have a potentially larger impact on understandings of life satisfaction across nations beyond the factors that were measured here.

## Conclusion

Constraints on generalisability notwithstanding, the present study provides an examination of the invariance of the SWLS across the largest number of nations to date (i.e., 65 nations here compared with a maximum of 26 nations previously) and across a large number of languages. Based on our results, we recommend that observed SWLS scores are not used for nation or language mean comparisons. Scholars intending to compare SWLS scores across nations and/or languages are advised to do so under conditions of partial or approximate invariance and with caution. Similar to Jang and colleagues [40], we also suggest that Items #1 and #3 are used as anchors when constructing CFA-based models across nations. Conversely, we suggest that more research is needed to understand sources of non-invariance in the SWLS, which the BINS was not set up to achieve. Doing so will not only help scholars better understand the meaning and manifestation of life satisfaction across nations, but will also aid practitioners in developing improved models of subjective well-being that, in turn, could inform public policy and population-level interventions aimed at improving life satisfaction [140,141].

## Supporting information

S1 Table Nations and associated sample sizes represented in the Body Image in Nature Survey (BINS). (DOCX)

S2 Table Invariance of cross-language survey presentation within Canada, China, Iceland, India, Philippines, and United Arab Emirates and of Using 5-Point and 7-Point response scales in the total sample. (DOCX)

S3 Table Results of the alignment method for national groups. (DOCX)

S4 Table Ranking of latent means (Cohen’s ds as compared to the United Kingdom) for national groups according to the partial scalar measurement model. (DOCX)

S5 Table Results of the alignment method for language. (DOCX)

S6 Table Ranking of latent means (Cohen’s ds as compared to English) for languages according to the partial scalar measurement model. (DOCX)

S1 Fig Distribution of reliability estimates in the national groups. (DOCX)

S2 Fig Heatmap of approximate invariant item parameters for national groups. (DOCX)

S3 Fig Scatterplots of the rankings of latent group means (national groups: Upper Panel; Languages: Lower Panel) according to the partial scalar measurement model and the alignment method. (DOCX)
