# Supplementary material for: Life satisfaction around the world: Measurement invariance of the Satisfaction With Life Scale (SWLS) across 65 nations, 40 languages, gender identities, and age groups
Source: PLoS One. 2025 Jan 22;20(1):e0313107. doi: 10.1371/journal.pone.0313107 (PMC11753666; doi:10.1371/journal.pone.0313107)
Supplement: S1 Table — (DOCX) [file pone.0313107.s001.docx]

**S1 Table. Nations and Associated Sample Sizes Represented in the Body Image in Nature Survey (BINS).**

| Nation | Data collection method | Survey language | Sample size | Ethics approval |
| --- | --- | --- | --- | --- |
| Argentina | Online | Spanish | 670 | University of Palermo |
| Australia | Online | English | 1,038 | Griffith University; University of the Sunshine Coast; Deakin University; University of Melbourne |
| Austria | Online | German | 1,279 | Exempted by Austrian national law |
| Bahrain | Online | Arabic | 441 | Royal College of Surgeons Ireland-Bahrain |
| Bangladesh | Online | Bangla | 460 | International University of Business Agriculture and Technology |
| Bosnia & Herzegovina* | Online | Bosnian | 406 | Czech Academy of Sciences |
| Brazil | Online | Portuguese | 1,462 | Centro de Capacitação Física do Exército |
| Bulgaria | Online | Bulgarian | 248 | Medical University of Plovdiv |
| Canada* | Online | English | 336 | St. Mary’s University |
|  | Online | French | 806 | Université du Québec en Outaouais and Université du Québec à Chicoutimi |
| Chile | Online | Spanish | 422 | Universidad Adolfo Ibáñez |
| China | Online | Cantonese | 409 | Exempt by Hong Kong regional law |
|  | Online | English | 349 | Lingnan University |
|  | Online | Mandarin | 1,231 | National Centre for International Research on Green Optoelectrics |
| Colombia* | Online | Spanish | 793 | University of Wrocław |
| Croatia | Online | Croatian | 898 | University of Zadar |
| Cyprus | Online | Greek | 363 | Neapolis University Pafos |
| Czechia* | Online | Czech | 700 | Czech Academy of Sciences |
| Ecuador | Online | Spanish | 863 | Universidad Regional Amazónica Ikiam |
| Egypt | Online | Arabic | 1,281 | Menoufia University |
|  | Paper-and-pencil | Arabic | 346 | Exempt by Egyptian national law |
| Estonia* | Online | Estonian | 449 | Exempt by Estonian national law |
| France | Online | French | 562 | Aix-Marseille University |
| Germany | Online | German | 620 | Medical School Hamburg |
| Ghana | Paper-and-pencil | English | 434 | Exempt by Ghanaian national law |
| Greece* | Online | Greek | 556 | Anglia Ruskin University |
| Hungary | Online | Hungarian | 654 | University of Miskolc |
| Iceland | Online | English | 1,149 | Exempt by Icelandic national law |
|  | Online | Icelandic | 432 | National Bioethics Committee of Iceland |
| India | Online | Hindi | 1,145 | Yes |
|  | Paper-and-pencil | Hindi | 519 | University of Allahabad; International Centre for Psychological Counselling and Social Research |
|  | Online | Tamil | 376 | International Centre for Psychological Counselling and Social Research |
| Indonesia | Online | Indonesian | 292 | Islamic University of Indonesia |
| Iran | Online | Farsi | 1,318 | Tarbiat Modares University; Tarbiat Modares University; Islamic Azad University of Bandar Gaz; Payam Noor University of Tehran |
| Iraq* | Paper-and-pencil | Arabic | 405 | Exempt by Iraqi national law |
| Ireland | Online | English | 351 | Anglia Ruskin University |
| Israel | Online | Hebrew | 493 | The Academic College of Tel Aviv-Yaffo |
| Italy | Online | Italian | 2,307 | Sapienza University of Rome |
| Japan | Online | Japanese | 360 | University of Tsukuba |
| Kazakhstan | Online | Russian | 380 | Al-Arabi Kazakh National University |
| Latvia | Online | Latvian | 827 | Exempt by Latvian national law |
| Lebanon | Online | Arabic | 1,295 | Lebanese American University; Holy Spirit University of Kaslik |
| Lithuania | Online | Lithuanian | 491 | Lithuanian Sports University |
| Malaysia | Online | Malay | 1,193 | Perdana University |
| Malta | Online | English | 347 | University of Malta |
| Nepal | Paper-and-pencil | Nepali | 353 | Exempt by Nepali national law |
| Netherlands | Online | Dutch | 1,004 | University of Groningen; Maastricht University |
| Nigeria | Paper-and-pencil | English | 1,274 | Ekiti State University; University of Ibadan |
| Norway* | Online | Norwegian | 360 | University of South-Eastern Norway; University of Agder |
| Pakistan | Online | English | 267 | University of Sargodha |
| Palestine* | Online | Arabic | 401 | al-Quds University |
| Philippines | Online | English | 350 | De La Salle University |
|  | Online | Tagalog | 504 | The Education University of Hong Kong |
| Poland | Online | Polish | 1,954 | University of Wrocław; University of Gdansk; Marie Curie-Skłodowska University; Poznan University of Physical Education |
| Portugal | Paper-and-pencil | Portuguese | 363 | University of Porto |
| Romania | Online | Romanian | 1,292 | West University of Timişoara |
|  | Paper-and-pencil | Romanian | 527 | Alexandru Ioan Cuza University |
| Russia* | Online | Russian | 206 | University of Hertfordshire |
| Saudi Arabia* | Online | Arabic | 380 | The University of Western Australia |
| Serbia | Online | Serbian | 650 | University of Novi Sad; University of Belgrade |
| Slovakia* | Online | Slovak | 814 | The University of Presov |
| Slovenia | Online | Slovene | 452 | University of Maribor |
| South Africa* | Online | English | 318 | Harvard University |
| South Korea* | Online | Korean | 381 | Handong Global University |
| Spain | Online | Spanish | 347 | Pontifical University of Salamanca |
|  | Paper-and-pencil | Spanish | 919 | University of Valencia; University of Almeria |
| Switzerland | Online | German | 377 | Exempt by Swiss national law |
| Taiwan* | Online | Mandarin | 529 | National Chung Cheng University |
| Thailand | Online | Thai | 3,275 | Thammasat University |
| Tunisia | Online | Arabic | 374 | University of Sousse |
| Türkiye | Online | Turkish | 2,518 | Munzur University; Koç University |
| Ukraine* | Online | Russian | 141 | University of Hertfordshire |
| United Arab Emirates* | Online | Arabic | 204 | United Arab Emirates University |
|  | Online | English | 904 | Middlesex University Dubai |
| United Kingdom | Online | English | 1,243 | Anglia Ruskin University |
| United States of America | Online | English | 2,531 | The Ohio State University; Clemson University; Chapman University; Virginia Wesleyan University; University of Missouri; Utah State University |

*Notes*. * Nations utilising a 5-point response scale in the SWLS in some or all languages the survey was presented in.
