# Supplementary material for: Life satisfaction around the world: Measurement invariance of the Satisfaction With Life Scale (SWLS) across 65 nations, 40 languages, gender identities, and age groups
Source: PLoS One. 2025 Jan 22;20(1):e0313107. doi: 10.1371/journal.pone.0313107 (PMC11753666; doi:10.1371/journal.pone.0313107)
Supplement: S2 Table — (DOCX) [file pone.0313107.s002.docx]

**S2 Table. Invariance of Cross-Language Survey Presentation within Canada, China, Iceland, India, Philippines, and United Arab Emirates and of Using 5-Point and 7-Point Response Scales in the Total Sample.**

|  |  |  |  |  |  |  | Model comparisons | | | | |
| --- | --- | --- | --- | --- | --- | --- | --- | --- | --- | --- | --- |
| Country (languages) | χ^2^(*df*) | CFI | TLI | RMSEA | 90% CI | SRMR | ΔCFI | ΔRMSEA | Configural | Metric |  |
| Canada (English vs. French) | |  |  |  |  |  |  |  |  |  |  |
| Configural invariance | 19.75(8) | .994 | .985 | .051 | [.023, .079] | .014 |  |  |  |  |  |
| Metric invariance | 31.29(12) | .990 | .984 | .053 | [.031, .076] | .043 | .004 | .002 | 11.74(4)* |  |  |
| Scalar invariance | 59.18(16) | .978 | .972 | .069 | [.051, .088] | .070 | .012 | .016 | 41.22(8)*** | 29.60(4)*** |  |
| China (Cantonese vs. English vs. Mandarin) | | |  |  |  |  |  |  |  |  |  |
| Configural invariance | 11.98(12) | 1.000 | 1.000 | .000 | [.000, .039] | .008 |  |  |  |  |  |
| Metric invariance | 24.04(20) | .999 | .998 | .017 | [.000, .039] | .032 | .001 | .017 | 12.84(8) |  |  |
| Scalar invariance | 96.55(28) | .981 | .979 | .061 | [.048, .074] | .054 | .018 | .044 | 95.91(16)*** | 86.95(8)*** |  |
| Iceland (English vs. Icelandic) | |  |  |  |  |  |  |  |  |  | |
| Configural invariance | 34.62(8) | .985 | .961 | .065 | [.044, .088] | .019 |  |  |  |  | |
| Metric invariance | 54.01(12) | .976 | .959 | .067 | [.049, .085] | .047 | .009 | .002 | 20.31(4)*** |  | |
| Scalar invariance | 154.70(16) | .920 | .900 | .105 | [.090, .120] | .085 | .056 | .038 | 155.74(8)*** | 149.73(4)*** | |
| India (Hindi vs. Tamil) |  |  |  |  |  |  |  |  |  |  | |
| Configural invariance | 18.73(8) | .996 | .990 | .036 | [.015, .058] | .012 |  |  |  |  | |
| Metric invariance | 44.68(12) | .988 | .980 | .052 | [.036, .068] | .042 | .008 | .016 | 27.58(4)*** |  | |
| Scalar invariance | 85.17(16) | .974 | .968 | .065 | [.052, .079] | .058 | .014 | .013 | 72.53(8)*** | 46.76(4)*** | |
| Philippines (English vs. Tagalog) | |  |  |  |  |  |  |  |  |  | |
| Configural invariance | 55.86(8) | .965 | .911 | .118 | [.090, .149] | .025 |  |  |  |  | |
| Metric invariance | 84.49(12) | .946 | .910 | .119 | [.096, .143] | .070 | .019 | .001 | 28.98(4)*** |  | |
| Scalar invariance | 169.33(16) | .886 | .858 | .150 | [.130, .171] | .106 | .060 | .031 | 138.23(8)*** | 114.76(4)*** | |
| United Arab Emirates (Arabic vs. English) | | |  |  |  |  |  |  |  |  | |
| Configural invariance | 40.76(8) | .978 | .945 | .086 | [.061, .113] | .032 |  |  |  |  | |
| Metric invariance | 66.31(12) | .963 | .939 | .090 | [.070, .112] | .058 | .015 | .004 | 25.93(4)*** |  | |
| Scalar invariance | 180.37(16) | .889 | .862 | .136 | [.119, .154] | .082 | .074 | .046 | 150.86(8)*** | 137.01(4)*** | |
| Total sample: 5-point vs. 7-point response scales | | |  |  |  |  |  |  |  |  | |
| Configural invariance | 474.36(8) | .994 | .984 | .045 | [.042, .049] | .011 |  |  |  |  | |
| Metric invariance | 552.64(12) | .993 | .988 | .040 | [.037, .043] | .014 | .001 | -.005 | 26.69(4)*** |  | |
| Scalar invariance | 832.71(16) | .989 | .986 | .042 | [.040, .045] | .021 | .004 | .002 | 326.04(8)*** | 313.30(4)*** | |

* *p* < .05, ** *p* < .01, *** *p* < .001.
