# Supplementary material for: Life satisfaction around the world: Measurement invariance of the Satisfaction With Life Scale (SWLS) across 65 nations, 40 languages, gender identities, and age groups
Source: PLoS One. 2025 Jan 22;20(1):e0313107. doi: 10.1371/journal.pone.0313107 (PMC11753666; doi:10.1371/journal.pone.0313107)
Supplement: S3 Table — (DOCX) [file pone.0313107.s003.docx]

**S3 Table. Results of the Alignment Method for National Groups.**

| Parameter and item | Estimate | *R*^2^ | Approximate measurement invariance holds for groups **(number of groups)** |
| --- | --- | --- | --- |
| Loadings |  |  |  |
| Item #1 | 1.17 | .756 | 2 4 5 6 7 9 10 11 12 13 14 15 16 17 18 21 22 24 25 26 27 28 29 30 32 33 34 35 36 37 38 39 40 41 43 44 45 46 47 48 49 51 52 53 54 55 56 57 58 59 60 61 62 63 64 65 66 67 68 69 70 71 72 **(63)** |
| Item #2 | 1.20 | .328 | 1 3 4 5 6 7 9 11 12 14 15 16 17 18 19 20 21 23 24 25 27 28 29 30 32 33 35 36 37 38 39 40 41 42 43 45 46 47 49 51 52 53 54 55 56 57 58 60 61 62 63 64 69 70 71 **(55)** |
| Item #3 | 1.31 | .000 | 1 2 3 4 5 6 7 10 12 13 14 15 16 17 18 19 21 23 24 25 26 27 28 29 30 32 33 34 36 37 38 39 40 41 43 44 45 46 47 48 49 50 51 52 53 54 55 56 57 58 59 60 61 62 64 65 66 67 68 69 70 71 72 **(63)** |
| Item #4 | 1.17 | .840 | 1 2 3 4 5 6 7 8 9 10 11 12 13 14 15 16 17 18 19 20 21 22 23 24 25 26 27 28 29 30 31 32 34 35 36 37 38 39 40 41 42 43 44 45 46 47 49 50 51 52 53 54 55 56 57 58 59 60 61 62 63 64 65 66 67 68 69 70 71 **(69)** |
| Item #5 | 1.14 | .070 | 1 3 5 6 7 8 9 10 11 12 13 14 15 16 17 18 19 20 22 23 24 25 26 27 28 30 32 33 34 36 37 38 39 40 41 42 43 44 45 46 47 48 50 51 52 53 54 57 58 59 60 61 63 64 65 66 69 70 71 72 **(60)** |
| Intercepts |  |  |  |
| Item #1 | 4.39 | .571 | 5 6 7 10 16 17 18 21 23 24 26 27 28 29 30 32 34 36 37 41 46 48 49 50 51 52 53 54 57 59 60 61 62 63 64 70 71 72 **(38)** |
| Item #2 | 4.77 | .809 | 3 5 6 9 10 11 12 13 15 18 21 22 24 25 27 28 29 31 32 33 34 35 37 38 40 41 43 45 46 47 49 54 55 57 59 60 61 62 63 65 66 67 69 70 **(44)** |
| Item #3 | 4.93 | .721 | 3 5 6 10 12 15 16 17 19 20 21 22 23 24 25 26 27 28 29 30 31 32 33 34 37 38 39 40 41 43 46 48 49 50 53 54 55 56 57 60 61 62 64 65 66 70 72 **(47)** |
| Item #4 | 4.83 | .576 | 2 3 5 6 10 12 14 17 19 21 22 25 27 28 29 32 34 35 36 37 38 42 43 45 46 47 48 52 53 56 58 59 62 63 64 65 66 67 68 70 72 **(41)** |
| Item #5 | 4.19 | .480 | 2 5 6 9 12 15 16 17 21 23 24 25 27 28 29 30 31 32 33 35 37 38 40 42 43 44 46 49 51 53 54 56 57 59 61 62 63 64 65 69 72 **(41)** |
| Average invariance index | | .523 |  |
| All loadings invariant | |  | 5 6 7 12 14 15 16 17 18 24 25 27 28 30 32 36 37 38 39 40 41 43 45 46 47 51 52 53 54 57 58 60 61 64 69 70 71 **(37)** |
| All intercepts invariant | |  | 5 6 21 27 28 29 32 37 46 62 72 **(11)** |
| All item parameters invariant | |  | 5 6 27 28 32 37 46 **(7)** |

*Note*. The fixed alignment method with the UK as anchor was used. Estimates represent weighted unstandardized average values across invariant groups. Group numbers represent: 1 = United Kingdom, 2 = Romania, 3 = United States of America, 4 = Hungary, 5 = Philippines (English), 6 = Lithuania, 7 = Netherlands, 8 = Iran, 9 = Lebanon, 10 = Argentina, 11 = Egypt, 12 = Austria, 13 = Malaysia, 14 = Thailand, 15 = China (English), 16 = Israel, 17 = Switzerland, 18 = Bahrain, 19 = Australia, 20 = Nigeria, 21 = Taiwan, 22 = Iraq, 23 = Saudi Arabia, 24 = Slovakia, 25 = Germany, 26 = Estonia, 27 = Canada (English), 28 = Slovenia, 29 = Japan, 30 = Iceland (English), 31 = India (Hindi), 32 = Cyprus, 33 = Ecuador, 34 = Colombia, 35 = Spain, 36 = South Korea, 37 = Serbia, 38 = Portugal, 39 = Türkiye, 40 = Ghana, 41 = Norway, 42 = Bangladesh, 43 = Bulgaria, 44 = Poland, 45 = Tunisia, 46 = United Arab Emirates (English), 47 = Palestine, 48 = Italy, 49 = Kazakhstan, 50 = Brazil, 51 = Latvia, 52 = Greece, 53 = Bosnia & Herzegovina, 54 = Chile, 55 = Nepal, 56 = Czechia, 57 = Malta, 58 = Indonesia, 59 = France, 60 = Russia, 61 = Ukraine, 62 = Pakistan, 63 = Croatia, 64 = Ireland, 65 = South Africa, 66 = China (Cantonese), 67 = China (Mandarin), 68 = Canada (French), 69 = Iceland (Icelandic), 70 = India (Tamil), 71 = Philippines (Tagalog), 72 = United Arab Emirates (Arabic). Group numbers 46 and 72 were not used
