# Supplementary material for: Life satisfaction around the world: Measurement invariance of the Satisfaction With Life Scale (SWLS) across 65 nations, 40 languages, gender identities, and age groups
Source: PLoS One. 2025 Jan 22;20(1):e0313107. doi: 10.1371/journal.pone.0313107 (PMC11753666; doi:10.1371/journal.pone.0313107)
Supplement: S4 Table — (DOCX) [file pone.0313107.s004.docx]

**S4 Table. Ranking of Latent Means (Cohen’s ds as Compared to the United Kingdom) for National Groups According to the Partial Scalar Measurement Model.**

| Rank | National group | Cohen’s *d* |
| --- | --- | --- |
| 1 | Canada (French) | 1.440 |
| 2 | Israel | 1.427 |
| 3 | Bosnia & Herzegovina | 1.247 |
| 4 | Iceland (English) | 1.237 |
| 5 | Ghana | 1.233 |
| 6 | Brazil | 1.213 |
| 7 | Slovenia | 1.186 |
| 8 | Spain | 1.165 |
| 9 | Croatia | 1.136 |
| 10 | Bulgaria | 1.098 |
| 11 | Iran | 1.094 |
| 12 | Chile | 1.081 |
| 13 | India (Hindi) | 1.077 |
| 14 | Colombia | 1.074 |
| 15 | Egypt | 1.074 |
| 16 | Serbia | 1.072 |
| 17 | Malaysia | 1.058 |
| 18 | Pakistan | 1.044 |
| 19 | Norway | 1.043 |
| 20 | Nepal | 1.042 |
| 21 | Romania | 1.039 |
| 22 | Iceland (Icelandic) | 1.002 |
| 23 | Netherlands | 0.998 |
| 24 | China (Cantonese) | 0.952 |
| 25 | Kazakhstan | 0.944 |
| 26 | Argentina | 0.938 |
| 27 | Cyprus | 0.917 |
| 28 | Hungary | 0.910 |
| 29 | Palestine | 0.900 |
| 30 | Philippines (English) | 0.878 |
| 31 | Bangladesh | 0.874 |
| 32 | Tunisia | 0.873 |
| 33 | Estonia | 0.870 |
| 34 | China (Mandarin) | 0.864 |
| 35 | India (Tamil) | 0.863 |
| 36 | Czechia | 0.857 |
| 37 | South Africa | 0.849 |
| 38 | Philippines (Tagalog) | 0.793 |
| 39 | UAE (English) | 0.793 |
| 40 | Lithuania | 0.772 |
| 41 | Greece | 0.757 |
| 42 | Portugal | 0.743 |
| 43 | South Korea | 0.729 |
| 44 | Switzerland | 0.718 |
| 45 | France | 0.717 |
| 46 | Indonesia | 0.710 |
| 47 | Malta | 0.702 |
| 48 | Slovakia | 0.698 |
| 49 | Thailand | 0.688 |
| 50 | Lebanon | 0.677 |
| 51 | Canada (English) | 0.657 |
| 52 | Poland | 0.636 |
| 53 | Austria | 0.575 |
| 54 | Latvia | 0.536 |
| 55 | Saudi Arabia | 0.525 |
| 56 | Ecuador | 0.514 |
| 57 | Türkiye | 0.502 |
| 58 | Bahrain | 0.497 |
| 59 | Italy | 0.451 |
| 60 | USA | 0.352 |
| 61 | Australia | 0.332 |
| 62 | Ireland | 0.309 |
| 63 | China (English) | 0.280 |
| 64 | Germany | 0.258 |
| 65 | Russia | 0.170 |
| 66 | United Kingdom | 0.000 |
| 67 | Taiwan | -0.048 |
| 68 | Ukraine | -0.113 |
