# Supplementary material for: Life satisfaction around the world: Measurement invariance of the Satisfaction With Life Scale (SWLS) across 65 nations, 40 languages, gender identities, and age groups
Source: PLoS One. 2025 Jan 22;20(1):e0313107. doi: 10.1371/journal.pone.0313107 (PMC11753666; doi:10.1371/journal.pone.0313107)
Supplement: S5 Table — (DOCX) [file pone.0313107.s005.docx]

**S5 Table. Results of the Alignment Method for Language.**

| Parameter and item | Estimate | *R*^2^ | Approximate measurement invariance holds for groups **(number of groups)** |
| --- | --- | --- | --- |
| Loadings |  |  |  |
| Item #1 | 1.35 | .715 | 1 3 4 5 6 7 8 10 11 12 13 14 15 16 17 18 20 21 22 23  25 26 27 28 29 31 32 34 35 36 37 38 39 41 42 **(38)** |
| Item #2 | 1.19 | .676 | 1 3 4 5 7 9 10 11 12 13 15 17 18 19 20 21 24 27 28 31  32 34 35 36 37 38 39 41 42 **(29)** |
| Item #3 | 1.46 | .582 | 1 2 3 5 8 9 10 11 12 13 14 15 16 17 18 20 21 22 23 24  25 26 28 29 30 31 32 34 35 36 37 38 39 41 **(34)** |
| Item #4 | 1.25 | .741 | 1 3 4 5 6 9 10 11 12 13 14 15 16 17 18 19 20 21 22 23  24 25 26 27 28 29 31 32 34 35 36 37 38 39 41 42 **(36)** |
| Item #5 | 1.33 | .121 | 3 4 5 6 7 8 9 10 12 13 15 16 17 20 21 22 23 25 26 27  28 29 30 32 34 35 36 37 39 41 42 **(31)** |
| Intercepts |  |  |  |
| Item #1 | 4.27 | .532 | 2 4 5 10 13 15 16 17 18 20 22 26 28 30 31 32 34 35 37 42 **(20)** |
| Item #2 | 4.59 | .842 | 1 3 5 7 8 11 17 18 19 21 22 23 26 28 34 36 38 41 42 **(19)** |
| Item #3 | 4.60 | .788 | 4 10 11 12 13 16 17 18 19 20 26 28 29 34 35 36 37 38 42 **(19)** |
| Item #4 | 4.70 | .656 | 2 5 6 9 14 17 18 20 21 23 25 27 28 30 32 34 37 39 41 42 **(20)** |
| Item #5 | 3.89 | .479 | 1 2 5 6 7 10 12 13 15 16 18 19 21 22 23 25 26 27 28 29 35 36 37 39 42 **(25)** |
| Average invariance index | | .613 |  |
| All loadings invariant | |  | 3 5 10 12 13 15 17 20 21 28 32 34 35 36 37 39 41 **(17)** |
| All intercepts invariant | |  | 18 28 42 **(3)** |
| All item parameters invariant | |  | 28 **(1)** |

*Note*. The fixed alignment method with English as anchor was used. Estimates represent weighted unstandardised average values across invariant groups. Group numbers represent: 1 = English, 2 = Romanian, 3 = Hungarian, 4 = Tagalog, 5 = Lithuanian, 6 = Farsi, 7 = Arabic, 8 = Spanish, 9 = German, 10 = Dutch, 11 = Malaysian Malay, 12 = Thai, 13 = Hebrew, 14 = Mandarin, 15 = Slovakian, 16 = Estonian, 17 = Slovenian, 18 = Japanese, 19 = Hindi, 20 = Greek, 21 = Serbian, 22 = Portuguese, 23 = Cantonese, 24 = Turkish, 25 = French, 26 = Norwegian, 27 = Bangla, 28 = Bulgarian, 29 = Polish, 30 = Italian, 31 = Russian, 32 = Korean, 33 = Brazil Portuguese, 34 = Tamil, 35 = Latvian, 36 = Icelandic, 37 = Bosnian, 38 = Nepali, 39 = Czech, 40 = Maltese, 41 = Indonesian, 42 = Croatian. Respondents from Brazil filled out the survey in Portuguese and respondents from Malta in English. Hence, only 40 languages were investigated in the present study and group numbers 33 and 40 were not used.
