# Supplementary material for: Life satisfaction around the world: Measurement invariance of the Satisfaction With Life Scale (SWLS) across 65 nations, 40 languages, gender identities, and age groups
Source: PLoS One. 2025 Jan 22;20(1):e0313107. doi: 10.1371/journal.pone.0313107 (PMC11753666; doi:10.1371/journal.pone.0313107)
Supplement: S6 Table — (DOCX) [file pone.0313107.s006.docx]

**S6 Table. Ranking of Latent Means (Cohen’s ds as Compared to English) for Languages According to the Partial Scalar Measurement Model.**

| Rank | Language | Cohen’s *d* |
| --- | --- | --- |
| 1 | Hebrew | 0.629 |
| 2 | Bosnian | 0.482 |
| 3 | Slovenian | 0.462 |
| 4 | Bulgarian | 0.431 |
| 5 | French | 0.418 |
| 6 | Croatian | 0.408 |
| 7 | Malaysian Malay | 0.378 |
| 8 | Portuguese | 0.376 |
| 9 | Hindi | 0.371 |
| 10 | Nepali | 0.360 |
| 11 | Serbian | 0.358 |
| 12 | Norwegian | 0.344 |
| 13 | Icelandic | 0.331 |
| 14 | Farsi | 0.316 |
| 15 | Romanian | 0.315 |
| 16 | Dutch | 0.309 |
| 17 | Cantonese | 0.265 |
| 18 | Spanish | 0.265 |
| 19 | Bangla | 0.246 |
| 20 | Hungarian | 0.246 |
| 21 | Estonian | 0.199 |
| 22 | Arabic | 0.191 |
| 23 | Tamil | 0.178 |
| 24 | Tagalog | 0.174 |
| 25 | Lithuanian | 0.163 |
| 26 | Czech | 0.153 |
| 27 | Greek | 0.140 |
| 28 | Korean | 0.072 |
| 29 | Slovakian | 0.047 |
| 30 | Indonesian | 0.045 |
| 31 | Thai | 0.011 |
| 32 | Polish | 0.010 |
| 33 | English | 0.000 |
| 34 | Mandarin | -0.035 |
| 35 | Latvian | -0.068 |
| 36 | Turkish | -0.101 |
| 37 | German | -0.110 |
| 38 | Russian | -0.111 |
| 39 | Italian | -0.157 |
| 40 | Japanese | -0.728 |
