# Supplementary material for: Life satisfaction around the world: Measurement invariance of the Satisfaction With Life Scale (SWLS) across 65 nations, 40 languages, gender identities, and age groups
Source: PLoS One. 2025 Jan 22;20(1):e0313107. doi: 10.1371/journal.pone.0313107 (PMC11753666; doi:10.1371/journal.pone.0313107)
Supplement: S1 Fig — (DOCX) [file pone.0313107.s007.docx]

**Fig. S1. Distribution of Reliability Estimates in the National Groups.**

**
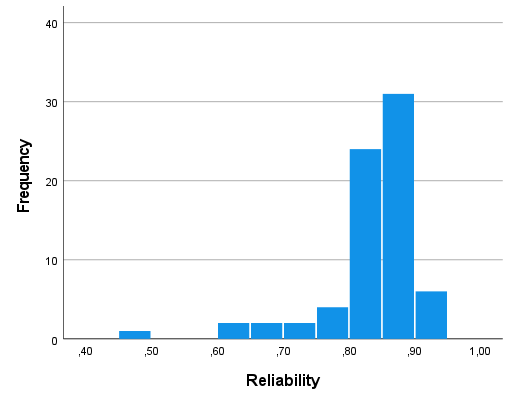
**

*Note*. The lowest value was .47 (United Arab Emirates [Arabic]) and the next higher value was .64 (India [Tamil]).
