# Supplementary material for: Life satisfaction around the world: Measurement invariance of the Satisfaction With Life Scale (SWLS) across 65 nations, 40 languages, gender identities, and age groups
Source: PLoS One. 2025 Jan 22;20(1):e0313107. doi: 10.1371/journal.pone.0313107 (PMC11753666; doi:10.1371/journal.pone.0313107)
Supplement: S2 Fig — (DOCX) [file pone.0313107.s008.docx]

**Fig. S2. Heatmap of Approximate Invariant Item Parameters for National Groups.**

**
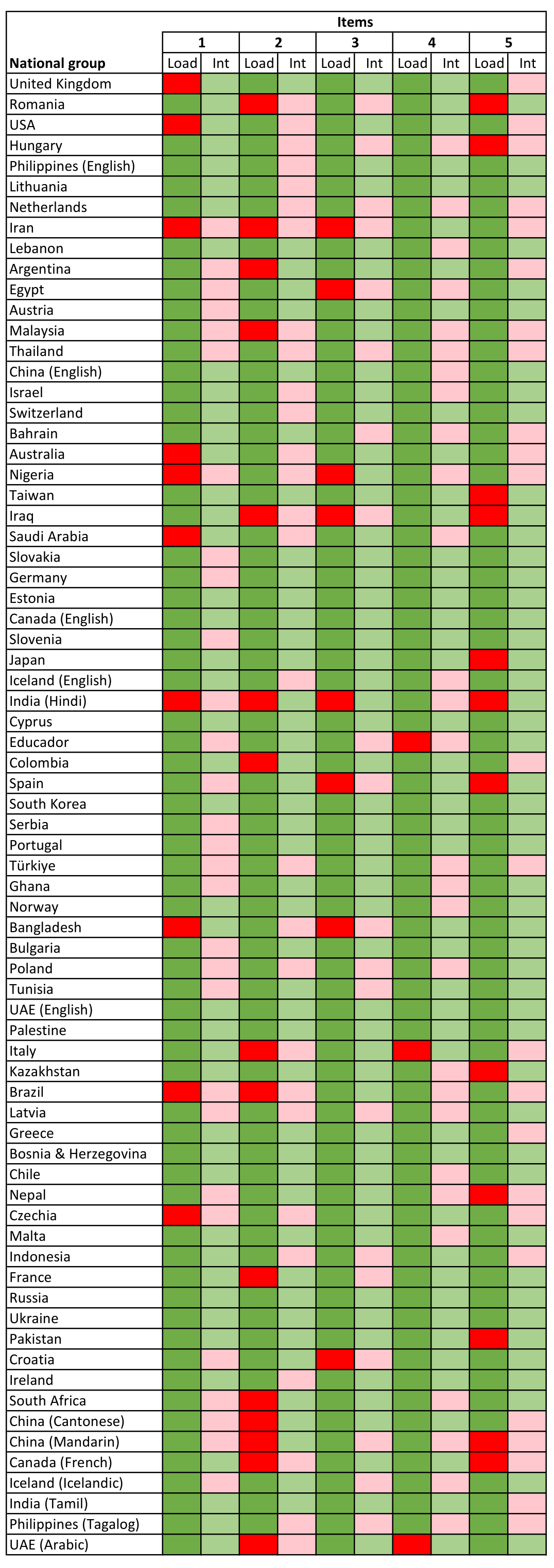
**

*Note*. Green signifies approximate invariance, red non-invariance (item loadings: high colour saturation; item intercepts: low colour saturation).
