# Supplementary material for: Life satisfaction around the world: Measurement invariance of the Satisfaction With Life Scale (SWLS) across 65 nations, 40 languages, gender identities, and age groups
Source: PLoS One. 2025 Jan 22;20(1):e0313107. doi: 10.1371/journal.pone.0313107 (PMC11753666; doi:10.1371/journal.pone.0313107)

**Fig. S3. Scatterplots of the Rankings of Latent Group Means (National Groups: Upper Panel; Languages: Lower Panel) According to the Partial Scalar Measurement Model and the Alignment Method.**


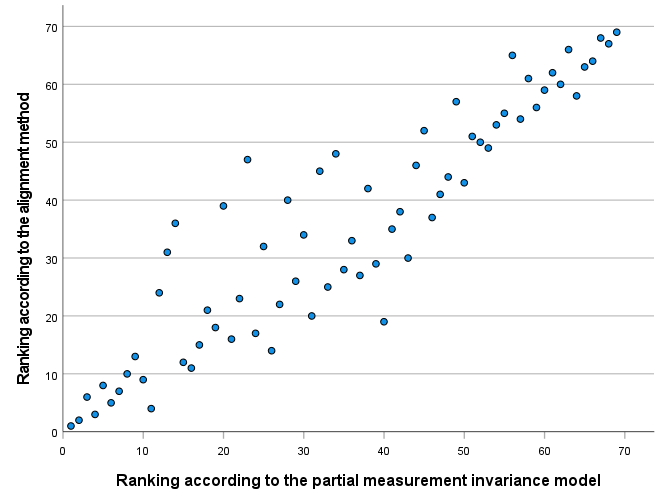


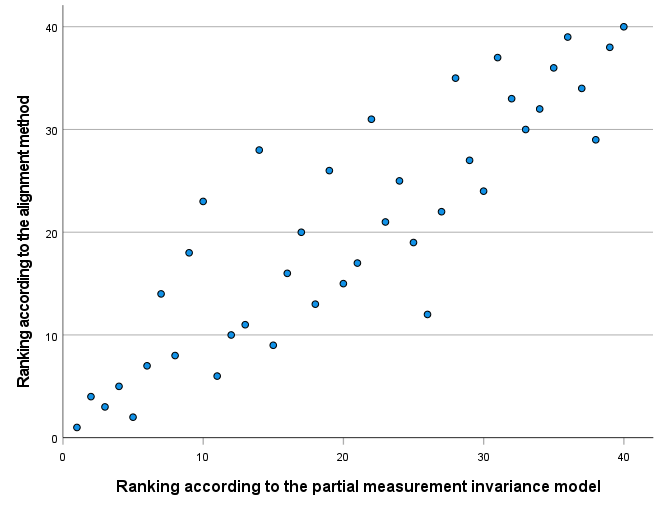

Supplement: S3 Fig — (DOCX) [file pone.0313107.s009.docx]
